# Safety, tolerability, pharmacodynamic and wellbeing effects of SPL026 (dimethyltryptamine fumarate) in healthy participants: a randomized, placebo-controlled phase 1 trial

**DOI:** 10.3389/fpsyt.2023.1305796

**Published:** 2024-01-11

**Authors:** Ellen James, David Erritzoe, Tiffanie Benway, Zelah Joel, Christopher Timmermann, Meghan Good, Claudio Agnorelli, Brandon M. Weiss, Tommaso Barba, Graham Campbell, Michelle Baker Jones, Charlotte Hughes, Helen Topping, Malcolm Boyce, Carol Routledge

**Affiliations:** ^1^Small Pharma Ltd., London, United Kingdom; ^2^The Centre for Psychedelic Research, Department of Brain Sciences, Faculty of Medicine, Imperial College London, London, United Kingdom; ^3^Hammersmith Medicines Research, London, United Kingdom

**Keywords:** dimethyltryptamine, major depressive disorder, pharmacodynamic, psychedelic, safety, tolerability

## Abstract

**Background:**

Due to their potential impact on mood and wellbeing there has been increasing interest in the potential of serotonergic psychedelics such as N,N-dimethyltryptamine (DMT) in the treatment of major depressive disorder (MDD).

**Aim:**

The aim of Part A of this study was to evaluate the safety, tolerability, pharmacokinetics (PK) and pharmacodynamic (PD) profile of escalating doses of SPL026 (DMT fumarate) in psychedelic-naïve healthy participants to determine a dose for administration to patients with MDD in the subsequent Phase 2a part of the trial (Part B: not presented in this manuscript).

**Methods:**

In the Phase 1, randomized, double-blind, placebo-controlled, parallel-group, single dose-escalation trial, psychedelic-naïve participants were randomized to placebo (*n* = 8) or four different escalating doses [9, 12, 17 and 21.5 mg intravenously (IV)] of SPL026 (*n* = 6 for each dose) together with psychological support from 2 therapy team members. PK and acute (immediately following dosing experience) psychometric measures [including mystical experience questionnaire (MEQ), ego dissolution inventory (EDI), and intensity rating visual analogue scale (IRVAS)] were determined. Additional endpoints were measured as longer-term change from baseline to days 8, 15, 30 and 90. These measures included the Warwick and Edinburgh mental wellbeing scale and Spielberger’s state-trait anxiety inventory.

**Results:**

SPL026 was well tolerated, with an acceptable safety profile, with no serious adverse events. There was some evidence of a correlation between maximum plasma concentration and increased IRVAS, MEQ, and EDI scores. These trends are likely to require confirmation in a larger sample size. Using the analysis of the safety, tolerability, PD, PK results, doses of 21.5 mg SPL026 were the most likely to provide an intense, tolerated experience.

**Conclusion:**

Based on the data obtained from this part of the trial, a dose of 21.5 mg SPL026 given as a 2-phase IV infusion over 10 min (6 mg/5 min and 15.5 mg/5 min) was selected as the dose to be taken into patients in Part B (to be presented in a future manuscript).

**Clinical trial registration:**www.clinicaltrials.gov, identifier NCT04673383; https://www.clinicaltrialsregister.eu, identifier 2020-000251-13; https://www.isrctn.com/, identifier ISRCTN63465876.

## 1 Introduction

Psychedelic drugs such as N,N-dimethyltryptamine (DMT), lysergic acid diethylamide, and psilocybin have played an important role in medicine, religious ceremonies and sociocultural rituals by indigenous peoples in South America and elsewhere, for thousands of years (1). In particular, DMT has been consumed for centuries as the principal hallucinogen within the psychedelic brew ayahuasca (2), where the other components (harmines, harmalines and other molecules) render DMT orally bioavailable by inhibiting the enzyme monoamine oxidase A (3). Previous evidence from ayahuasca use suggests increased feelings of wellbeing and improved health outcomes in both naïve and regular users (4–7). Over the last 50 years, DMT has been studied in healthy volunteers where it has produced a range of psychedelic effects such as those on perceptions, emotion, mood alterations and anxiety (2, 8–14). These effects are seen within 2 min after intravenous (IV) bolus administration and then resolve within 30 min (14).

The serotonergic system has long been implicated in the pathogenesis of major depressive disorder (MDD) (15–17). The actions of serotonin on target cells, including glutamatergic and gamma-aminobutyric acid (GABAergic) neurons, are mediated by a large family of 5HT receptors (including the 5HT1 & 5HT2 subfamilies) (18). Like other classical psychedelic compounds, DMT acts via modulation of the serotonergic system, in particular the 5HT2A receptor (19–22), which is believed to be primarily responsible for its hallucinogenic and interoceptive effects (23). DMT also binds with high affinity to other serotonin receptors, some of which may play key roles in depression, particularly 5HT1A (20), and 5HT2C (22).

Due to their potential impact on mood and wellbeing there has been increasing interest in the potential of serotonergic psychedelics such as DMT in the treatment of MDD (7, 24–28). In studies of patients with recurrent MDD or treatment-resistant depression (TRD), a single dose of ayahuasca was associated with significant and clinically relevant improvements in depressive symptoms (7, 27). Data from a small preliminary report in 6 participants with recurrent depressive symptoms indicated an 82% reduction in depressive symptom scale scores, with no tolerability issues, after a single dose of ayahuasca (29). However, none of these studies have formally (regulatory approved, blinded and placebo-controlled) evaluated the efficacy and safety of DMT in isolation from ayahuasca which contains several other molecules that could contribute to its therapeutic effect, although some relevant data has been published (14, 28, 30). In one study, DMT at IV doses of 0.05, 0.1, 0.2, and 0.4 mg/kg dose-dependently increased blood pressure and heart rate in 11 psychedelic-experienced volunteers (10, 11). More recently, changes in brain activity were found to be correlated with the psychedelic experience in 13 psychedelic-experienced individuals and also highlighted the similarity of a DMT experience to a near-death experience (14, 30). There are some concerns that psychedelic substances can produce adverse psychological effects, including fear, paranoia and anxiety (31), as well as modest cardiovascular effects. Therefore, assessment of safety and tolerability of the psychedelic experience of DMT at doses targeted for therapeutic efficacy in future clinical trials in both psychedelic-naïve and psychedelic-experienced participants is important if they are to be evaluated in a therapeutic context.

The current study evaluated the safety, tolerability, pharmacokinetics (PK) and pharmacodynamics (PD) of four different single ascending IV doses of SPL026 (DMT fumarate) compared with placebo in psychedelic-naïve healthy volunteers. The PK data, including information on the infusion regimen, has been published elsewhere (19). This study is the Phase 1 component of a Phase 1/2a trial and was designed to determine a dose of SPL026 for administration to patients with MDD in the subsequent Phase 2a part of the trial (Part B; not presented within this manuscript).

## 2 Materials and methods

### 2.1 Study outline

The Phase 1 study was a randomized, double-blind, placebo-controlled, parallel-group dose-escalation trial (ClinicalTrials.gov: NCT04673383; EudraCT: 2020-000251-13; ISRCTN: ISRCTN63465876) (the Phase 2a part of this study will be reported separately). The study was conducted to meet criteria of European Medicines Agency (EMA) guidelines and Good Clinical Practice and was approved by the UK Medicines and Healthcare Products Regulatory Agency (MHRA) and London Brent ethics committee.

Although this was not a first-in-human study, EMA guidelines for risk identification and risk mitigation were followed (32), together with scientific advice from the Medicines and Healthcare Products Regulatory Agency (UK). Additionally, the study was designed in accordance with published guidelines regarding safe clinical assessment of psychedelic substances in humans (33).

### 2.2 Study population

Male and female participants were required to be aged 25 years and above, with no previous exposure to serotonergic psychedelic substances, and registered with a general practitioner within the UK. The age limit for healthy participants was 25 years, because some mental health disorders (such as schizophrenia) most commonly manifest before age 25. Participants were eligible for the trial if they had a negative drugs of abuse screen at day-1; a history of nicotine use was acceptable (≤ to 10 cigarettes daily) but participants had to refrain from smoking when in the clinical unit (day-1 to day 2). In addition, participants had to refrain from taking cannabis 24 h prior to study visits. Participants were also required to have a body mass index 18.0–30.9 kg/m^2^, no clinically relevant physical findings, electrocardiogram (ECG) or laboratory values at the screening visit. Participants with a current or past diagnosis of a mental health disorder as defined by the American Psychiatric Association Diagnostic and Statistical Manual of Mental Disorders, fifth edition (DSM-5), or a history of suicide attempts, or a first- or second-degree relative with a psychotic disorder or bipolar disorder were excluded from the study.

Psychedelic-naïve participants were intentionally selected for this trial because SPL026 will ultimately be evaluated in patients with MDD, many of whom will be psychedelic-naïve.

### 2.3 Study design and procedures

**Figure 1:**
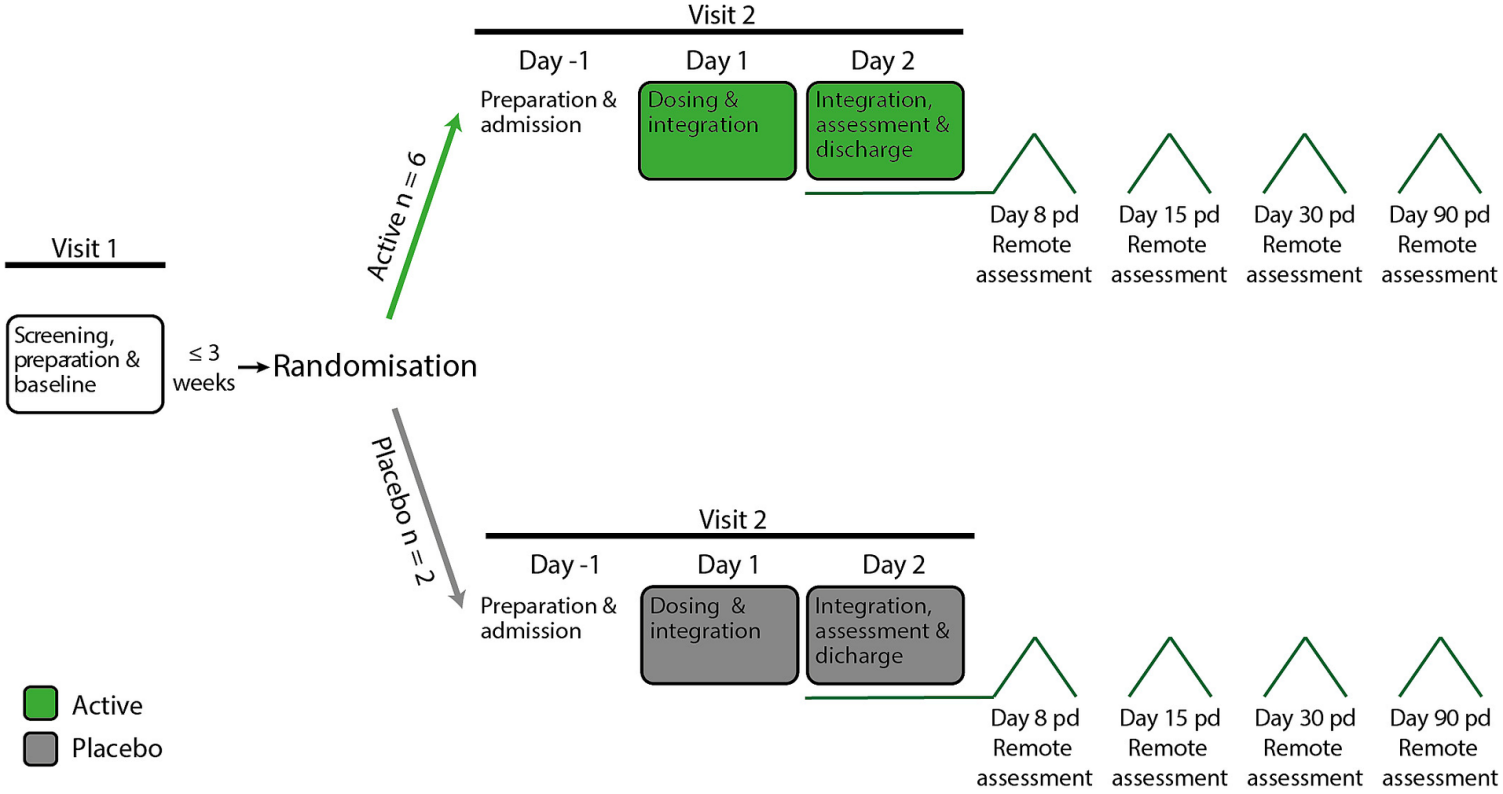
Phase 1 study design remote assessment: telephone or video call. Pd, part dose.

At the screening visit (Figure 1), participants provided written informed consent and participated in a structured psychiatric interview with the study psychiatrist using the Mini-International Neuropsychiatric Interview (Adult M.I.N.I. Screen 7.0.2 for DSM-5; 8/8/16 version).

All participants underwent preparation sessions that included advice on what to expect and how to respond to the psychedelic experience and a bespoke visualization guide, which acts as a means of exploring and processing the psychedelic experience (further detail will be provided in a future publication), at screening and again the day prior to and the morning of study drug administration. Also at screening, participants completed a series of rating scales (described in detail below), including the Beck scale for suicidal ideation (BSS) (34), Warwick-Edinburgh mental wellbeing scales (WEMWBS) (35) and Spielberger’s state-trait anxiety inventory TRAIT subscale (STAI-T) (36). Other screening assessments included physical examination, assessment of vital signs, 12-lead ECG, clinical laboratory tests, and review of prior and concomitant medications.

Participants were admitted to the clinical pharmacology unit [Hammersmith Medicines Research (HMR), London, United Kingdom] the day before study drug administration, to be familiarized with the setting and study staff. Participants completed baseline assessments and underwent a preparation session where they received advice on what to expect and how to respond to the psychedelic experience (the therapy component will be described in detail in a separate publication). On the day of treatment, pre-dose assessments were completed. Study drug was administered in a room set up according to best practice principles for psychedelic studies (33), including soft lighting, soft furnishings, music and photographs/art depicting scenes of nature. Each dosing room contained a dedicated therapist team (either 2 therapists, or 1 psychiatrist and 1 therapist) and additional personnel (clinical staff for supervising dosing and PK sampling). Final day 1 assessments were completed at 240 min post-dose. Participants remained in the unit to day 2.

Participants were divided into 4 cohorts of 8; each participant received a single dose of SPL026 or placebo by a continuous 10 min IV infusion split into 2 phases (Table 1). A single cannula was inserted in the forearm vein with two separate syringes and two separate syringe pumps, connected by a 3-way tap in order to provide different infusion rates for the two continuous phases of infusion: Phase 1 of the infusion: an infusion of 6 mg SPL026 (or equivalent volume of placebo) was given over 5 min with the intention to bring the participants to the verge or early stages of a psychedelic experience; Phase 2 of the infusion: the infusion was given over 5 min with the objective of achieving a sufficient psychedelic experience at higher dose levels. One of the key aims of a slower infusion rate (versus a bolus IV dose) was to take subjects gradually and gently into what may be an intense psychedelic experience, which in turn may enhance tolerability to the psychedelic experience in patients with MDD who may be psychedelic-naïve.

**Table 1:** Planned study groups and doses.

Group	Planned total dose^a^	Planned duration of IV infusion		Actual total dose	Actual duration of IV infusion	
1	9 mg SPL026	Phase 1:	6 mg SPL026 over 5 min	9 mg SPL026	Phase 1:	6 mg SPL026 over 5 min
		Phase 2:	3 mg SPL026 over 5 min		Phase 2:	3 mg SPL026 over 5 min
2	12 mg SPL026	Phase 1:	6 mg SPL026 over 5 min	12 mg SPL026	Phase 1:	6 mg SPL026 over 5 min
		Phase 2:	6 mg SPL026 over 3 min		Phase 2:	6 mg SPL026 over 5 min
3	17 mg SPL026	Phase 1:	6 mg SPL026 over 5 min	17 mg SPL026	Phase 1:	6 mg SPL026 over 5 min
		Phase 2:	11 mg SPL026 over 4 min		Phase 2:	11 mg SPL026 over 5 min
4	21.5 mg SPL026	Phase 1:	6 mg SPL026 over 5 min	21.5 mg SPL026	Phase 1:	6 mg SPL026 over 5 min
		Phase 2:	15.5 mg SPL026 over 3 min		Phase 2:	15.5 mg SPL026 over 5 min
^b^5	20 mg SPL026	Phase 1:	10 mg SPL026 over 5 min	—	—	—
		Phase 2:	10 mg SPL026 over 5 min		—	—

DMT, dimethyltryptamine; IV, intravenous.

a Dose refers to free base DMT (not DMT fumarate unless otherwise stated). Participants received SPL026 as a continuous infusion in 2 phases with a total infusion duration of 6–11 min. Doses of SPL026 did not exceed 21.5 mg and planned doses were subject to change in light of data obtained in preceding dosing groups.

b Group 5 was optional.

At each dose level (cohort), up to 6 participants received SPL026 and 2 received matching placebo, with 48 h between the two sentinel participants of each cohort being dosed, and each of the remaining participants of that group being dosed. To maintain the blinded nature of the study, the 2 sentinel participants were randomized to ensure that 1 received active treatment and the other received placebo. Provided that the investigator and sponsor considered the safety and tolerability in the sentinel participants (up to and including day 2 procedures) were acceptable at the dose escalation meeting, the remaining participants were dosed as planned (1 participant per day).

The dose was increased in the subsequent cohort only if the safety, tolerability and PK of the previous dose were acceptable [with the aim of attaining a sufficient psychedelic experience with minimal somatic effects; defined as the cohort mean of ≥60% on the mystical experience questionnaire (MEQ) from all participants on active treatment]. After dosing, participants were required to remain in the clinical unit overnight for further psychological and safety evaluations on day 2. They were then discharged the next day dependent on satisfactory outcomes of these assessments. Follow-up was conducted at regular intervals by phone or video call for up to 3 months after study treatment.

The dose selection for the Phase 2a study (Part B; proof of concept in patients with MDD, not presented in this manuscript) took into account all the data from each of the dose cohorts in Phase 1. The dose was selected based on no safety or tolerability concerns and one that elicited a psychedelic experience in subjects on active treatment which gave a mean average of ≥60% of the maximum possible score on the MEQ. In addition, the combined average (increase in) WEMWBS and (reduction in) STAI-T score (change from baseline at 2 weeks after dosing) was not significantly lower than the scores in any other cohort.

### 2.4 Psychological support

The therapeutic approach taken in the Phase 1 study was a time-limited, relational psychotherapeutic framework, based around the concept of psychological flexibility, and built on the foundation of a person’s life history. It is a process centered around the subjective experience, that prioritizes therapist presence and attunement to the individual’s psychedelic experience. This is based on the accept, connect, embody (ACE) model of psychological flexibility (37) with the addition of relational and transpersonal elements. The support was provided by 2 members of the therapy team (qualified and trained therapists and psychiatrists) and consisted of a preparation session at screening, day-1 and immediately prior to dosing, support during dosing and integration sessions immediately after dosing and on the day after dosing.

### 2.5 Integration and tolerability

During the post-treatment integration session, participants were encouraged to discuss their experience (including tolerability) with the therapy team. The first session was conducted as a semi-structured conversation, in order to capture the experience in as much detail as participants were able to provide; the therapist could ask open-ended questions to clarify elements of the experience, but no interpretations were attempted. From Cohort 2 onwards, this session included the question: “*Do you wish you had not gone through that experience*?.” Cohort 1 were not asked this question as it was added to the study protocol as an amendment after they had been dosed.

### 2.6 Drug manufacturing

SPL026 drug substance and drug product were manufactured in the UK in accordance with Good Manufacturing Practice, with 2.5 mg/mL DMT free base in 10 mL aqueous sterile solution; placebo consisted of the same ingredients and volume with the exception of the active substance. Both active and placebo treatments were prepared ensuring that the pH was 4.0 for both. Active and placebo treatments were packaged and labeled by an unblinded pharmacist at the HMR pharmacy and were identical in appearance and administered in the same volume. The study participants, investigators, study therapists, study psychiatrists and clinical and medical monitors remained blinded until the end of the study.

### 2.7 Study endpoints and assessments

The primary safety endpoints of the study included treatment-emergent adverse events (TEAEs) (collected using a non-leading question, such as “How are you feeling?” and also spontaneously reported by participants) and serious adverse events (SAEs) (Medical Dictionary for Regulatory Activities v. 24.1); suicidality (change from baseline to post-dose on day 8 as measured by the BSS, repeated on days 15, 30 and 90; a higher score indicates greater suicidality); and clinical laboratory tests. Other secondary safety assessments included vital signs, physical examination and any reactions at the sites at which the drug infusion catheter and the PK sampling catheter were inserted. Laboratory values were considered to be of potential clinical importance (PCI) if they were outside the published normal ranges and changed from baseline (day-1) by more than the predefined limit. Investigators judged whether a change in vital signs was of PCI on the basis of whether the value caused concern, necessitated medical intervention or if it resulted in the participant being withdrawn or given concomitant medication. Events associated with the psychedelic experience were not recorded as TEAEs because they form part of the intended therapeutic mechanism of action of SPL026.

The definitive question in the post-treatment integration session regarding tolerability was: “*Do you wish you had not gone through that experience*?,” and participant intolerance was defined as a positive response to this question. Further participant experience evaluation was quantified using the intensity rating visual analogue scale (IRVAS), which asks: “*How intense was the experience*?” (14). The IRVAS was carried out by the participant and the therapist or psychiatrist after integration and the scores of the two were averaged to get an overall IRVAS score. The therapist and psychiatrist used the information provided by the participant during the integration session to provide their IRVAS score. For this scale, 0 indicates that the experience was not intense at all and 100 indicates an extremely intense experience. Secondary endpoints included the pharmacokinetic and pharmacodynamic endpoints; the latter used a number of questionnaires and scales. Scales completed by participants after the psychedelic experience had ended, but before integration, were: MEQ, ego dissolution inventory (EDI), emotional breakthrough inventory (EBI), challenging experience questionnaire (CEQ), the 5-dimension altered states of consciousness questionnaire (5D-ASCQ), a set of 25 additional visual analog scales (VAS), and the metaphysical experience questionnaire (MPEQ).

Self-reported secondary endpoints were measured as longer-term change from baseline (screening) to days 8, 15, 30 and 90. These measures included: WEMWBS, STAI-T, profile of mood states (POMS), brief experiential avoidance questionnaire (BEAQ), gratitude questionnaire (GQ-6), Snaith Hamilton anhedonia pleasure scale (SHAPS), flourishing scale (FS-8), life orientation test (LOT), meaning in life questionnaire (MLQ), brief resilience scale (BRS), dysfunctional attitude scale (DAS), Barrett impulsivity scale (BIS), social connectedness scale (SCS), the CompACT scale, openness enriched Big Five inventory (BFI), Watts connectedness scale (WCS), and psychological insight scale (PIS).

A 64-lead active electrode system (actiCHamp Plus, Brain Products GmbH, Gilching, Germany) was used to record electroencephalography (EEG) activity for a maximum period of 90 min before until 4 h after dosing. EEG recording was paused on cessation of the psychedelic experience until the post-dose integration and interview were completed, while ensuring that certain EEG tasks were completed (to be published elsewhere).

### 2.8 Statistical analyses

As this was a Phase 1 study with a small number of planned participants, no sample size calculation was undertaken. The sequential single ascending dose escalation group design was based on the Association of the British Pharmaceutical Industry Phase 1 clinical trial guideline example (38). The safety population comprised all participants who received at least 1 dose of study treatment, the PD population comprised all subjects who received at least 1 dose of the study drug and had at least 1 available PD measurement, and the PK population comprised participants who received at least 1 dose of study treatment and for whom a blood sample had been analyzed. Actual sampling times were used to derive PK parameters and missing data were not imputed. Plasma concentrations and PK parameters were summarized by treatment, using descriptive statistics. Descriptive statistics were derived using SAS 9.4 or higher, including mean, standard deviation (SD), median, minimum and maximum values. No formal statistical testing was predefined in the statistical analysis plan for any of the outcomes (safety, PK or PD) except for analyses of variance (ANOVAs) with a single factor of dose were performed on measures of wellbeing and STAI-T.

### 2.9 Dose and exposure-response analyses

Analyses were performed to explore the relationship between the results of the PD scales and dose, and maximum plasma concentration (*C*_max_) following IV administration of placebo or SPL026 (9, 12, 17 and 21.5 mg IV). Details of the PK analyses have previously been reported (19).

The analysis of the relationship between single acute (immediately after dosing experience had ended) measures (MEQ, EDI, EBI, CEQ, 5D-ASCQ, and VAS of the different features of the experience) and *C*_max_ values was performed using Pearson’s correlation to test for a linear relationship (reported using the Pearson’s *R* coefficient and *p*-value with *α* < 0.05), whereas a polynomial regression model (Formula: Score = *β*2 × *C*_max_^2^ + *β*1 × *C*_max_ + *β*) was used to test for a quadratic relationship (reported using the *F*-value and *p*-value with *α* < 0.05). For these models, the dependent variable was the average score on the different psychometric questionnaires and the independent variable was the *C*_max_ value of those participants who received a dose of SPL026 (placebo were assigned a *C*_max_ value of 0).

The analysis of long-term measures’ differences relative to drug dosage was performed using a linear mixed-effects model. The dependent variable was the scores from the different psychometric questionnaires (POMS, BEAQ, GQ-6, SHAPS, FS-8, LOT, MLQ, BRS, DAS, BIS, SCS, CompACT, BFI, WCS, PIS), the independent variables were the drug doses (placebo and SPL026 9, 12, 17, 21.5 mg IV) and the time points (baseline, and 8, 15, 30, and 90 days after drug exposure) plus their interaction, inserted as a fixed effect, and a random intercept was added for each participant. The analysis of long-term measures differences as moderated by *C*_max_ values was conducted using a linear mixed-effects model. The dependent variable was the scores on the different psychometric questionnaires, the independent variables were the *C*_max_ values of those participants who received a dose of SPL026 (*z*-scored by subtracting the mean to each value and dividing by the SD) and the time points plus their interaction, inserted as a fixed effect, and a random intercept was added for each participant. The analysis of the quadratic moderation effect of *C*_max_ on long-term measures was performed by adding to the previous model the squared *C*_max_ values as additional independent variables. Results of the linear mixed-effects models are reported using the chi-squared (*χ*^2^) statistics and *p*-value (with *α* < 0.05). For the report of interaction effects, the unstandardized coefficients (*B*) and *p*-values are reported (with *α* < 0.05).

To account for family-wise false discovery rate (FDR) inflation due to multiple comparisons, the *p*-values resulting from tests performed on acute (immediately after dosing experience had ended) measures and long-term measures were adjusted independently using the Benjamini–Hochberg adjustment (39). Results of the FDR correction are reported as “*p*-adjusted” in the main text and Supplementary material.

For analysis of WEMWBS and STAT-T as part of Phase 2a dose selection the combined average (increase in) WEMWBS and (reduction in) STAI-T score (change from baseline at 2 weeks after dose) should not be significantly lower than any for other cohorts, analyzed using ANOVA and Tukey’s multiple comparisons. Data from all participants (SPL026 and placebo) with data at baseline and Day 15 timepoints were included in the analysis. If data for one or more cohorts failed to meet the assumptions required for the ANOVA model, an equivalent non-parametric test was used. In addition, one-way ANOVAs for each WEMWBS timepoints were conducted comparing placebo and 9, 12, 17 and 21.5 mg SPL026.

## 3 Results

### 3.1 Participants

The Phase 1 study randomized 32 healthy, psychedelic-naïve participants [age range, 25–76 years; 8 (25%) females; 15 (47%) non-white] who received study treatment and 30 were evaluated for safety, tolerability and PD effects; participant demographics and disposition have previously been published in (19) and are summarized in Table 2. Of these, 8 participants were randomized to placebo (2 per dosing group) and 24 participants were randomized to SPL026 (6 per dosing group), all of whom also received psychological support. Data for participants receiving placebo were pooled from all dosing cohorts. Two participants (1 each from 9 mg and 17 mg SPL026 cohorts) had major protocol deviation dosing errors (9 mg participant had a cannula leak; 17 mg participant had a 4 min pause in dosing). As such, their data is excluded from the analysis.

**Table 2:** Demographics of the study participants (safety population).

Parameter	Placebo (*n* = 8)	SPL026 9 mg (*n* = 6)^a^	SPL026 12 mg (*n* = 6)^a^	SPL026 17 mg (*n* = 6)^a^	SPL026 21.5 mg (*n* = 6)^a^
**Age, years**					
Mean (SD)	31.9 (6.7)	34.3 (7.1)	34.5 (8.7)	43.0 (17.4)	40.0 (8.5)
Range	26–42	27–43	25–44	28–76	28–48
Male, *n*	7	5	2	5	5
Female, *n*	1	1	4	1	1
Race, *n*					
White	5	2	3	3	4
Asian	0	3	0	1	1
Black	1	0	1	1	1
Latin American	1	0	0	0	0
Mixed race	1	1	2	1	0
**Weight, kg**					
Mean (SD)	84.5 (4.8)	79.5 (14.2)	59.6 (5.5)	74.2 (12.8)	80.6 (7.9)
Range	75.9–92.3	63.0–98.9	51.6–65.7	61.8–90.2	66.4–89.4
**Body mass index, kg/m**^**2**^					
Mean (SD)	26.5 (2.0)	25.7 (3.0)	21.4 (2.3)	25.0 (4.0)	26.0 (2.4)
Range	24.0–29.8	23.1–30.6	19.0–24.1	20.0–30.1	23.6–29.8
Cigarettes^b^, *n* (daily)	0	0	0	0	1 (5 daily)
Alcohol^b^ (units/week)					
*n*	6	3	4	3	3
Mean (SD)	4.5 (3.15)	2.7 (1.15)	2.5 (0.58)	5.7 (5.69)	4.0 (2.00)

SD, standard deviation.

a Doses are free base dimethyltryptamine.

b Includes only those subjects who smoke/drink alcohol.

### 3.2 Safety

#### 3.2.1 Adverse events

A total of 25 participants (78%) experienced at least 1 TEAE: 6 in the pooled placebo group, 5 each in the 9 mg, 12 mg and 17 mg SPL026 cohorts and 4 in the 21.5 mg SPL026 cohort. Of these 25 participants, drug-related TEAEs were reported by 13 participants (41%) (Table 3): 1 each in the pooled placebo and 21.5 mg SPL026 cohorts, with 2, 4 and 5 participants reporting drug-related TEAEs in the 9, 12 and 17 mg SPL026 cohorts, respectively. There were no SAEs, no withdrawals due to TEAEs, and all TEAEs were rated as mild or moderate in severity. The most frequently reported TEAEs included catheter site reactions (at drug infusion catheter site and PK sampling catheter site) (41%), sleep disorder (16%) and headache (13%). At 1 h post-dose, there were no reported signs of erythema, induration, pain or tenderness at the injection site. Further details can be found in Good et al. (19).

**Table 3:** Summary of most frequently reported drug-related treatment emergent adverse events (safety population).

Number of participants with TEAEs system organ class	Pooled placebo (*N* = 8)	SPL026 9 mg^a^ (*N* = 6)	SPL026 12 mg^a^ (*N* = 6)	SPL026 17 mg^a^ (*N* = 6)	SPL026 21.5 mg^a^ (*N* = 6)	Total (*N* = 32)
Gastrointestinal disorders: total			2			2
Abdominal discomfort			1			1
Nausea			1			1
General disorders and administration site conditions: total	1	2	1	2	1	7
Catheter site pain		1	1			2
Catheter site related reaction					1	1
Infusion site pain	1			2		3
Infusion site reaction		1				1
Cardiac and vascular investigations: total				2		2
Heart rate increased				2		2
Nervous system disorders: total				3		3
Dizziness				1		1
Headache				2		2
Psychiatric disorders: total			5			5
Anxiety			1			1
Euphoric mood			2			2
Sleep disorder			2			2
Skin and subcutaneous tissue disorders: total				1		1
Cold sweat				1		1
Vascular disorders				2		2
Pallor				2		2
Overall total	1	2	8	10	1	22

TEAE, treatment-emergent adverse event.

a Doses are free base dimethyltryptamine.

#### 3.2.2 Tolerability

During the interview the psychiatrist asked a number of questions discussing the participant’s subjective psychedelic experience. The definitive question asked of the participants to assess tolerability was: “*Do you wish you had not gone through that experience*?” All participants from Cohort 2 onwards provided a negative response to this question indicating that SPL026 was well tolerated.

#### 3.2.3 Suicidality

The mean BSS score was zero for all dose cohorts at screening and prior to dosing, and remained at zero through 90 days of follow-up, with the exception of a mean score of 0.2 of a maximum of 10 (first 5 questions of BSS) at day 30 in the 12 mg SPL026 cohort. This score returned to zero at day 90. This mean change was attributable to a single participant who scored 1 (weak) rather than zero on the question which asked “My wish to die is.” They were followed up by the psychiatrist, who concluded this was an error as the subject had misunderstood the form and did not show signs of suicidality.

#### 3.2.4 Clinical laboratory assessment and vital signs

There were no changes in mean laboratory variables (clinical chemistry, hematology, and coagulation variables) that could reasonably be attributed to trial medication; the list of laboratory variables that were tested can be found in the Supplementary material. Participants with any laboratory value of PCI are summarized below. Two participants [1 receiving placebo and 1 receiving 12 mg SPL026 had 7.9 mmol/L total cholesterol (above normal limit of 7.2 mmol/L) and 3.3 mmol/L potassium (below normal limit of 3.5 mmol/L)], respectively. Two participants [2 receiving SPL026 (one at a dose of 12 mg and one at a dose of 17 mg) had a slight decrease in activated partial thromboplastin time– 21.1 s and 18.8 s, respectively (below reference range of 28–42 s)]. No change in laboratory value was considered to be clinically significant and there were no clinically significant physical examination findings.

Post-dose vital signs of PCI were recorded in the following number of participants: 2 (33.3%) after 9 mg; 4 (66.7%) after 12 mg; 3 (50.0%) after 17 mg; and 6 (100.0%) after 21.5 mg SPL026. There was evidence of a possible relationship between SPL026 dose and raised blood pressure, as follows. Post-dose systolic blood pressures of PCI were recorded in 2 (33.3%) participants after 9 mg; 4 (66.7%) after 12 mg; 3 (50.0%) after 17 mg; and 5 (83.8%) after 21.5 mg SPL026. The greatest increases in blood pressure were in the last cohort, with a maximum % change from baseline of 55% in one participant at 12 min post-dose (mean 25.7% change) (Table 4). Measurements were all above the reference interval (ranging 143–173 mmHg) except an isolated value (89 mmHg) that was below. Post-dose diastolic blood pressures of PCI were recorded in 2 (33.3%) participants after 9 mg; 3 (50.0%) after 12 mg; 1 (16.7%) after 17 mg; and 2 (33.3%) after 21.5 mg SPL026. All measurements were above the reference interval (91–110 mmHg). No blood pressures of PCI were recorded in participants who received placebo. All changes from baseline in systolic and diastolic blood pressure were observed at the 12 min post-dose time point, but these were transient and not sustained at 1 h post-dose.

**Table 4:** Systolic and diastolic blood pressure changes in participants receiving placebo or different doses of SPL026.^a^

Treatment	Planned relative time	Mean (SD)		Change from baseline (SD)		% change from baseline (SD)	
		DBP	SBP	DBP	SBP	DBP	SBP
Placebo (*N* = 8)	Pre-dose	67.38 (5.50)	122.88 (3.36)	—	—	—	—
	12 min	74.38 (6.99)	127.13 (8.39)	7.00 (7.80)	4.25 (7.30)	10.87 (12.20)	3.44 (5.89)
	60 min	69.63 (9.23)	122.25 (8.35)	2.25 (8.33)	−0.63 (6.86)	3.47 (12.76)	−0.54 (5.56)
	240 min	69.00 (5.40)	117.63 (4.00)	1.63 (8.45)	−5.25 (5.39)	3.15 (13.00)	−4.21 (4.24)
9 mg SPL026 (*N* = 5)	Pre-dose	74.60 (8.32)	118.80 (15.07)	—	—	—	—
	12 min	82.20 (11.65)	137.00 (18.40)	7.60 (11.59)	18.20 (8.81)	10.77 (14.85)	15.51 (7.88)
	60 min	72.80 (9.47)	122.20 (15.25)	−1.80 (7.09)	3.40 (2.88)	−2.26 (8.70)	2.90 (2.23)
	240 min	71.40 (10.50)	115.20 (12.64)	−3.20 (12.36)	−3.60 (7.16)	−3.54 (15.10)	−2.71 (5.94)
12 mg SPL026 (*N* = 6)	Pre-dose	76.17 (10.94)	116.00 (7.51)	—	—	—	—
	12 min	88.17 (16.53)	139.00 (21.19)	12.00 (14.38)	23.00 (16.15)	16.47 (19.48)	19.46 (13.78)
	60 min	75.33 (12.23)	116.00 (8.07)	−0.83 (4.67)	0.00 (3.29)	−1.12 (6.41)	0.00 (2.79)
	240 min	73.17 (11.65)	116.00 (12.39)	−3.00 (5.37)	0.00 (7.21)	−3.83 (7.22)	−0.15 (6.40)
17 mg SPL026 (*N* = 5)	Pre-dose	64.00 (10.56)	119.80 (12.60)	—	—	—	—
	12 min	70.40 (21.85)	133.00 (38.37)	6.40 (25.24)	13.20 (33.18)	13.40 (44.29)	10.43 (26.50)
	60 min	66.80 (6.30)	119.20 (11.80)	−3.60 (24.61)	−0.60 (12.07)	−7.67 (42.91)	0.04 (10.53)
	240 min	69.20 (9.20)	121.80 (11.14)	2.40 (3.58)	2.00 (10.65)	3.14 (5.18)	2.18 (9.47)
21.5 mg SPL026 (*N* = 6)	Pre-dose	71.00 (7.48)	120.33 (15.93)	—	—	—	—
	12 min	86.00 (10.43)	149.67 (14.40)	15.00 (8.65)	29.33 (13.84)	21.58 (13.11)	25.70 (16.05)
	60 min	74.00 (9.78)	115.17 (11.37)	3.00 (6.69)	−34.50 (15.35)	4.30 (9.23)	−22.62 (9.71)
	240 min	74.17 (11.43)	118.67 (15.92)	3.17 (8.84)	3.50 (7.09)	4.50 (12.00)	2.78 (6.24)

SD, standard deviation; SBP, systolic blood pressure; DBP, diastolic blood pressure.

a Dose is free base dimethyltryptamine.

There were no conclusive differences among treatment groups with respect to mean pulse rate or body temperature and no pulse rate or temperature measurements of PCI. No post-dose vital sign of PCI was considered by the investigator to be clinically significant.

### 3.3 Pharmacodynamics

No dose-response relationship was observed between *C*_max_ of the four SPL026 doses and PD measures, however, there was an SPL026-related relationship with placebo being different to the four active doses. For each measure presented below, first the dose-response relationship is explored, followed by the *C*_max_-response relationship.

#### 3.3.1 Mystical experience questionnaire

There was a clear SPL026-related effect on MEQ, which increased with dose: mean (range) MEQ was 2.29 (0.17–4.13) following 9 mg; 2.92 (0.43–4.60) following 12 mg; 3.74 (1.67–4.90) following 17 mg; and 3.00 (1.37–4.43) following 21.5 mg SPL026 administration; compared with 0.51 (0.00–2.60) following placebo administration.

The above findings suggest that complete mystical experiences were more frequent at higher doses (≥17 mg SPL026) than at lower doses of SPL026. However, complete mystical experiences, or average MEQ scores of ≥60% on all 4 scales, were recorded across all dose levels, in 9 participants: 1 following 9 mg; 3 following 12 mg; 3 following 17 mg; and 2 following 21.5 mg SPL026 administration. Average MEQ scores in those participants ranged between 82.6% and 98.0% (where 5 is 100%).

**Figure 2:**
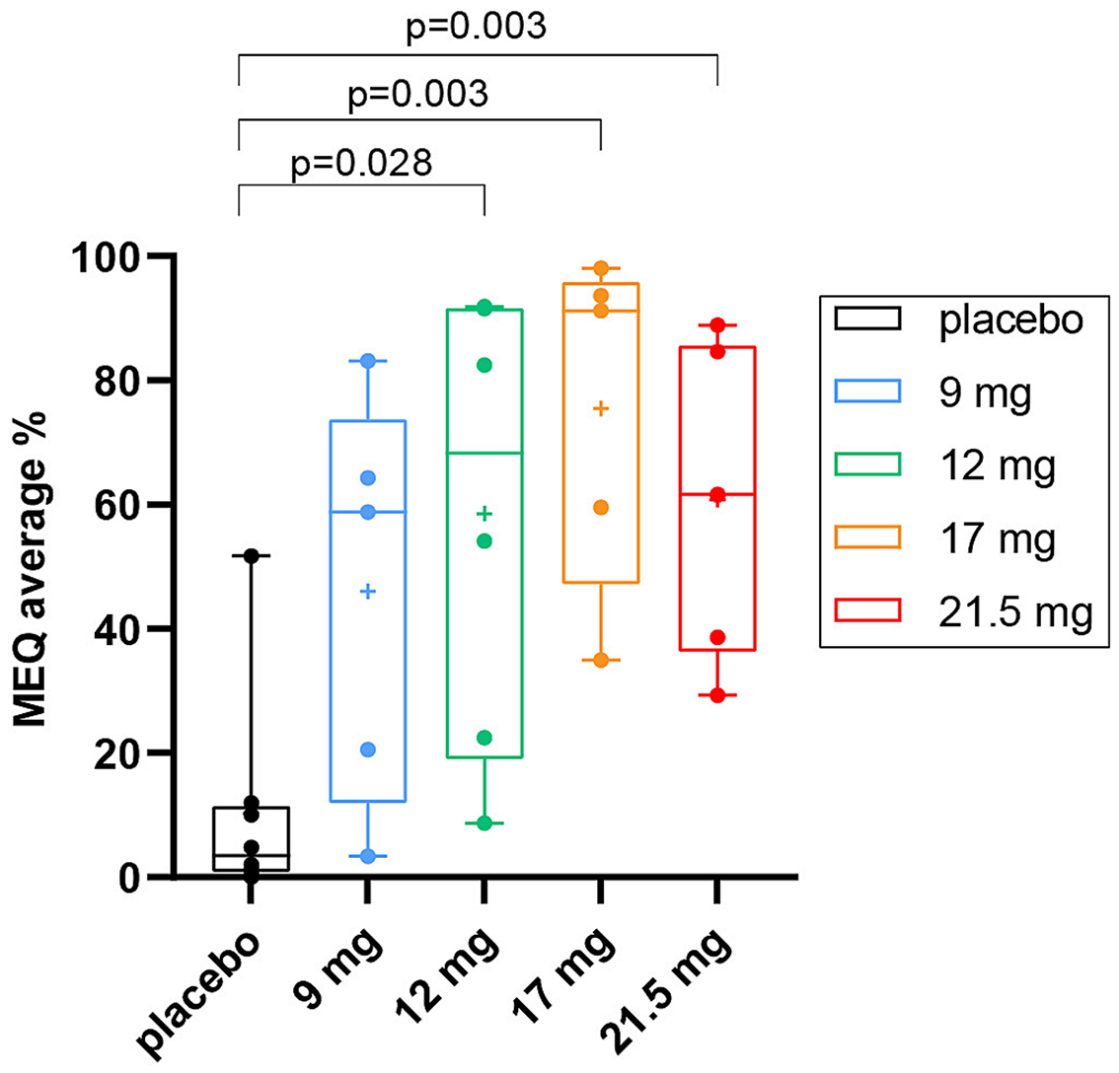
Average % MEQ scores box and whisker plots displaying group differences in MEQ scores. The average MEQ scores (as percentage of the maximum score of 5) are shown. Individual observations are shown as colored dots, the line through box is median and “+” is the mean. The *p*-values correspond to post-hoc results (Tukey-corrected for multiple comparisons). MEQ, mystical experience questionnaire.

The ANOVA revealed a significant difference in the average score in the MEQ across groups; *F* (df = 4, *N* = 30) = 5.324, *p*-adjusted = 0.016 (raw *p*-value = 0.003) (Supplementary Table S1). Post-hoc multiple comparisons showed that the average MEQ score was lower in the placebo group (*M* = 0.512 ± 0.87) as compared to 12 mg SPL026 (*M* = 2.92 ± 1.83, *p* = 0.027, Tukey correction), 17 mg SPL026 (*M* = 3.74 ± 1.40, *p* = 0.003, Tukey correction), and 21.5 mg SPL026 (M = 3 ± 1.23, *p* = 0.022, Tukey correction), but not to the 9 mg group (M = 2.29 ± 1.64, *p* = 0.199, Tukey correction) (Figure 2). However, no significant differences were observed in average MEQ among those receiving SPL026 doses (plus psychological support).

**Figure 3:**
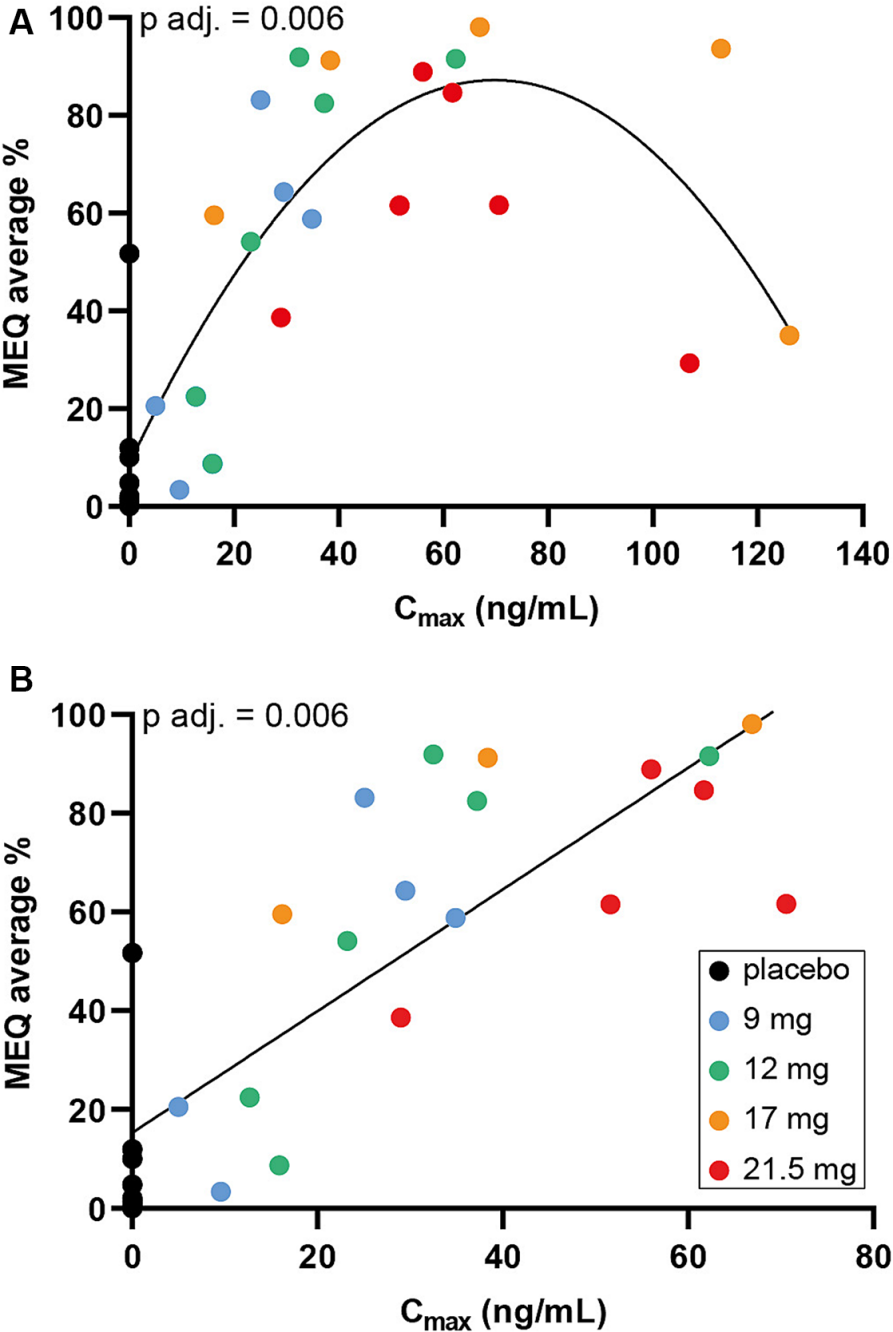
Maximum SPL026 plasma concentration versus MEQ score. **(A)** Scatter plot of SPL026 *C*_max_ and MEQ score. **(B)** Scatter plot of SPL026 *C*_max_ and MEQ excluding 3 participants with the highest *C*_max_ values (i.e., *C*_max_> 80 ng/mL). On the *x*-axis the *C*_max_ values are shown. On the *y*-axis the average scores on the different scales are shown. In the scatterplot, individual observations are shown as colored dots. The *p*-values correspond to **(A)** the quadratic regression analysis results after FDR correction and **(B)** the Pearson’s analysis after FDR correction, abbreviated as “*p*-adj.” *C*_max_, maximum plasma concentration; MEQ, mystical experience questionnaire.

Given a non-significant relationship between *C*_max_ and dose, a linear regression model was fitted to assess the predictive power of *C*_max_, to account for the subjective measures of the drug. There was a significant inverted U-shape relationship between *C*_max_ and the average scores on MEQ (*F* = 11.460, *p*-adjusted = 0.006) (raw *p*-value <0.001) (Figure 3A and Supplementary Table S1). Thus, progressively higher values of *C*_max_ predict higher scores in the relative subjective experience questionnaires until a certain threshold value, after which higher *C*_max_ levels are associated with lower scores. The observed effect was mainly driven by 3 participants with the highest *C*_max_ values (i.e., *C*_max_ > 80 ng/mL), 2 from the 17 mg SPL026 cohort and 1 from the 21.5 mg SPL026 cohort. Given the small sample size of observations at the high end of the *C*_max_ range, concerns over the reliability of the quadratic findings led us to test the linear relationship between *C*_max_ and drug experience while excluding these three observations with particularly high *C*_max_. For this analysis, a parametric Pearson’s correlation was used. Without the highest *C*_max_ participants, there was a significant positive correlation between *C*_max_ and MEQ (*R* = 0.71, *p*-adjusted = 0.006) (raw *p*-value <0.001) (Figure 3B).

#### 3.3.2 Intensity-rating visual analogue scale

Participants recorded a higher intensity of experience according to the IRVAS than the therapist at lower doses—following administration of 9 mg SPL026, the range in IRVAS was 3–54 (physician) compared with 9–100 (participant); the combined range IRVAS was 6–77 at this dose level. However, at doses of ≥12 mg SPL026, therapist- and participant-lead measurements were generally more similar.

**Figure 4:**
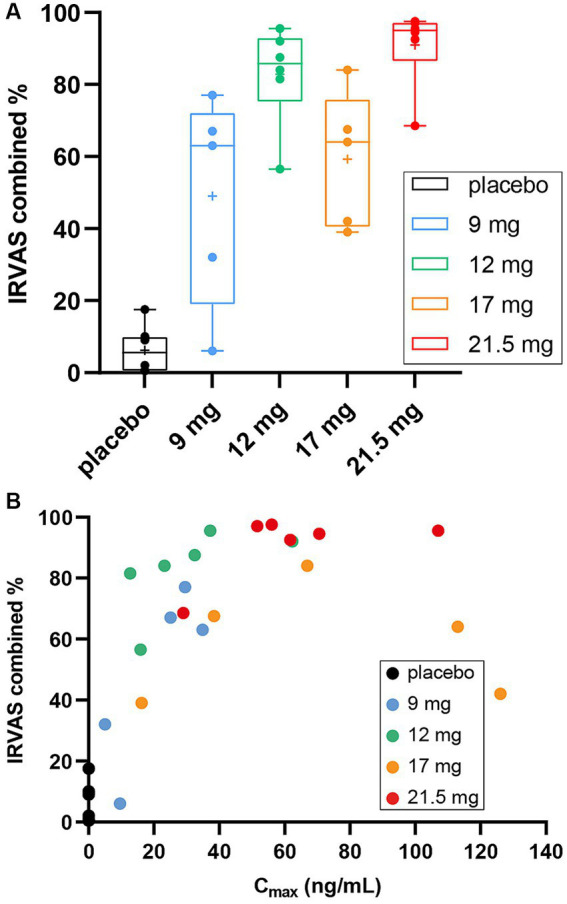
Combined IRVAS scores **(A)** box and whisker plots of mean combined IRVAS score. Individual observations are shown as colored dots, the line through box is median and “+” is the mean. **(B)** Scatter plot displaying *C*_max_ vs. combined IRVAS score. *C*_max_, maximum plasma concentration; IRVAS, intensity rating visual analogue scale.

There was a SPL026-related effect on combined IRVAS compared with placebo, which generally increased with dose: mean (range) combined IRVAS was 49.0 (6–77) after 9 mg; 82.8 (57–96) after 12 mg; 59.3 (39–84) after 17 mg; 90.9 (69–98) after 21.5 mg SPL026; and 6.2 (1–18) after placebo (Figure 4A). As with MEQ, there was a *C*_max_-response relationship, with 2 subjects from the SPL026 17 mg cohort reporting lower than expected intensity ratings, relative to their *C*_max_ (Figure 4B).

The ANOVA revealed a significant difference in the average score in the combined IRVAS across groups; F (df = 4, *N* = 30) = 9.9733, *p*-adjusted = 0.001 (raw *p*-value = <0.001). Post-hoc multiple comparisons showed significant differences between all comparisons, except for those receiving SPL026 9 mg vs. 17 mg, 12 mg vs. 17 mg and 12 mg vs. 21.5 mg (all participants also received psychological support: details are presented in Supplementary Table S2).

#### 3.3.3 Other acute measures taken immediately following the end of dosing experience

Further analysis of dose-response showed that the EDI score was significantly lower in the placebo group than in the 17 mg SPL026 cohort, but not in the other cohorts. The EBI score was significantly lower in the placebo cohort than in the 12 mg and 17 mg SPL026 cohorts, and the 17 mg SPL026 cohort had a significantly higher EBI score than the 9 mg and 21.5 mg SPL026 cohorts. There was no significant difference in the CEQ score across groups (only 2 subjects had average CEQ scores ≥0.5; maximum possible =1.0: 12 mg SPL026, CEQ 0.58; 17 mg SPL026, CEQ 0.89). For the 5D-ASCQ, the Oceanic boundlessness and visionary restructuralization dimension scores were significantly lower in the pooled placebo cohort than in some or all of the SPL026 cohorts (Supplementary Figure S1). There was a significant difference in several VAS of psychedelic experience across cohorts. Generally, the placebo group scored lower compared to the SPL026 cohorts, while the differences between SPL026 doses varied (Supplementary Tables S1, S2).

As with MEQ, there was a significant inverted U-shape relationship between *C*_max_ and the average scores on EDI (*F* = 7.219, *p*-adjusted = 0.021) (raw *p*-value = 0.005), EBI (*F* = 11.460, *p*-adjusted = 0.006) (raw *p*-value <0.001), and 5D-ASCQ dimensions of Oceanic boundlessness (*F* = 7.149, *p*-adjusted = 0.021) (raw *p*-value = 0.005), and Visionary restructuralization (*F* = 6.475, *p*-adjusted = 0.024) (raw *p*-value = 0.007) (Supplementary Figure S2). A similar result was observed for several VAS scores relative to psychedelic experience (Supplementary Table S2). Looking at the linear relationship between *C*_max_ and acute drug experience while excluding the three observations with particularly high *C*_max_, there was a significant positive correlation between *C*_max_ and EDI (*R* = 0.67, *p*-adjusted = 0.013) (raw *p*-value = 0.002), and 5D-ASCQ dimensions of oceanic boundlessness (*R* = 0.62, *p*-adjusted = 0.021) (raw *p*-value = 0.005), Visionary restructuralization (*R* = 0.61, *p*-adjusted = 0.021) (raw *p*-value = 0.006) (Supplementary Table S1 and Supplementary Figure S3). Also, a similar result was observed for several VAS scores relative to psychedelic experience (Supplementary Table S2).

#### 3.3.4 Wellbeing and anxiety scales

For mental wellbeing and anxiety assessments in the trial, the WEMBWBS and the STAI-T were used. The dose selected for the Phase 2a part of this study was required to meet the criteria that there were no significant differences between the mean scores for WEMBWBS and STAI-T taken from subjects in each cohort who received SPL026. A combined ANOVA was used to determine any differences between cohorts that may indicate there was a detrimental effect of SPL026 administration on the mental health of psychedelic-naïve healthy subjects (Table 5).

**Table 5:** Summary of ANOVA results for summed WEMWBS and STAI-T scores.

Source	df	Sum of squares	Mean square	*F* value	Pr > *F*
Model	3	41.59	13.86	0.20	0.8935
Error	27	1844.61	68.32	—	—
Corrected total	30	1886.19	—	—	—

ANOVA, analysis of variance; df, degrees of freedom; WEMWBS, Warwick-Edinburgh mental wellbeing scales; STAI-T; Spielberger’s state-trait anxiety inventory-TRAIT subscale.

To examine the effect of SPL026 on wellbeing, we assess participants before and at intervals after dosing (Supplementary Table S3). Baseline WEMWBS ranged between 48 and 70. As determined by ANOVA, there was no significant difference in WEMWBS data between SPL026 and placebo groups at all time points, and between-participant variability was high. There was no evidence for a relationship between WEMWBS score and *C*_max_ (not shown). STAI-T mean scores were similar at screening across dosing groups and timepoints (Supplementary Table S4). Post-dose mean changes from baseline, and between-participant variability was high in all cohorts. Hence, there was no evidence of a SPL026-related change in participants’ anxiety scores when compared with placebo.

#### 3.3.5 Other long-term measures

POMS, BEAQ, GQ-6, SHAPS, FS-8, LOT, MLQ, BRS, DAS, BIS, SCS, CompACT, BFI, MBS and WCS were assessed at multiple time points before and after administration of SPL026 and placebo. The effect of dose on psychometric variables was assessed using a linear mixed-effects model. Overall, no significant difference between groups for most of the outcomes analyzed, except for the Agreeableness domain of the BFI (Supplementary Table S5). When looking at the interaction of *C*_max_ with the long-term outcome measures, no statistically significant difference between groups was observed over time in most instances (i.e., no significant *C*_max_ × time interaction). The only significant interaction was found for the Agreeableness domain of the BFI questionnaire; *χ*^2^ (df = 1, *N* = 22) = 12.767, *p*-adjusted = 0.015 (raw *p*-value <0.001). In particular, *C*_max_ was significantly related to change in agreeableness, such that being higher in *C*_max_ by 1 SD was associated with an incremental decrease in Agreeableness by 0.20 units from baseline to day 90 after drug exposure (*B* = −0.197, *p* = 0.002).

## 4 Discussion

This is the first randomized, placebo-controlled study designed to formally evaluate the safety, tolerability, PD and PK profile of different IV doses of SPL026 (9 mg, 12 mg, 17 mg or 21.5 mg) in psychedelic-naïve healthy participants. This Phase 1 study demonstrated the safety, tolerability, and feasibility of delivering SPL026 via a two-phase slow IV infusion in order to slightly slow down the rise to an intense psychedelic experience. The aim of this was to enhance the tolerability of the elicited psychedelic experience in psychedelic-naïve healthy subjects in preparation for the next phase of the clinical trial (Phase 2a)—administering SPL026 to patients with MDD who may also be psychedelic-naïve.

At the doses administered in this trial, SPL026 had an acceptable safety profile; there were no SAEs and no apparent relationship between SPL026 dose and frequency of TEAEs, including cardiovascular TEAEs such as increased heart rate and blood pressure. No participants withdrew due to TEAEs, indicating that SPL026 administered IV over a 10 min infusion at these doses, in a supervised setting, was well-tolerated among psychedelic-naïve participants. These safety results are consistent with previous findings from smaller studies showing that DMT had a favorable safety and tolerability profile, although these studies were conducted in psychedelic-experienced healthy volunteers whereas the current study was conducted in subjects who were psychedelic-naïve. Strassman et al. (10, 11) previously reported neuroendocrine, cardiovascular, autonomic, and subjective effects of DMT (at doses of 0.05, 0.1, 0.2 and 0.4 mg/kg IV) in psychedelic-experienced volunteers. In these studies, DMT elevated blood pressure and heart rate but overall the experience was well tolerated up to doses of 21.5 mg (free base), a dose equivalent to 35 mg or ~0.5 mg/kg DMT fumarate. This was also the case when Strassman subsequently administered DMT at a dose of 0.3 mg/kg IV on four separate occasions over the course of 1 day to 13 psychedelic-experienced healthy participants (40) and Vogt subsequently administered DMT via a bolus injection and 30 min infusion (41). It has been suggested that psychedelic-experienced subjects may better tolerate DMT, particularly with respect to anticipatory anxiety, and therefore if the observed elevations in blood pressure and heart rate are due to anticipatory anxiety then differences between these previous studies and the current study may be predicted. Based on the analysis conducted within the present trial, there was no correlation between cardiovascular effects and plasma levels of DMT, in addition, there was no correlation between the intensity of psychedelic experience and cardiovascular effects. These data suggests that SPL026 may not have a direct effect on cardiovascular parameters but rather that the increases in these measures could be due to anticipatory anxiety that can occur as the participant experiences the initial subjective effects of DMT at the onset of infusion and before they are immersed in the psychedelic experience. This potential effect of psychedelics on anticipatory anxiety has been corroborated by other researchers evaluating the effects of psilocybin (42) and DMT (43) in healthy subjects.

Although psychedelics are generally well tolerated, as was found in the current study, their administration involves psychological risks. One of the key issues is a challenging experience illustrated by anxiety, fear/panic, dysphoria, and/or paranoia (33). These intense emotional experiences may lead to potentially dangerous and erratic behavior (33). When administered in a clinical setting, with psychological support from specially trained therapists, SPL026 was well tolerated in all participants, as no psychedelic-naïve healthy volunteer stated that they wished they had not gone through the experience, for every dose tested (the first cohort were not asked this question specifically, but the therapy team raised no concerns). The average consolidated intensity rating of the psychedelic experience (as measured using the MEQ), at each dose level up to 17 mg SPL026, was ≤85%. Increases in experience intensity were not associated with changes in tolerability at any dose of SPL026. This may also suggest that the slower IV infusion rate, and the resulting slower ascent to the psychedelic experience determine the good tolerability of SPL026. The psychological support framework used in this Phase 1 study will form the basis for the therapeutic approach when administering SPL026 to MDD patients. Given the low risk of psychological adverse events following administration of SPL026 in the current study, and the tolerability demonstrated here and in prior academic research studies and the favorable safety profile, the benefit-to-risk ratio of progressing SPL026 into efficacy studies in patient populations is deemed positive.

In terms of emotional wellbeing outcomes, the data demonstrate that there were no effects of SPL026 on the short- or long-term effect on any psychometric scale other than an increase in the Agreeableness dimension of the BFI, which measured changes in personality 3 months after dosing. The mean WEMWBS score at baseline was 61.4 (SD 6.77). The UK population scores show that the top 15% of scores taken from the general population range (from 60–70; possible scores 14–70) (44), showing that the study participants were within the high range for wellbeing at screening. The mean score across all participants at day 90 was 59.0 (SD 7.15), with no significant differences seen between baseline readings and treatment (active or placebo). This result, as well as the results seen for the other long-term measures in this study (other than BFI agreeableness) suggests that these study participants were very psychologically robust upon enrolling on the study, and/or that SPL026 neither improved nor impaired their psychological wellbeing. That there was no improvement in wellbeing or anxiety scores may indicate that the healthy participants in this study were already at the ceiling level. These outcomes were overall concordant with previous work evaluating the effects of DMT in other relevant populations. An exploratory pilot study in 10 subjects with a range of levels of previous psychedelic experience (3 healthy participants and 7 patients with MDD) showed that IV DMT was well-tolerated with no detrimental effects on wellbeing (28). A favorable safety profile was also seen in a more recent study of DMT given as a bolus followed by IV infusion over 30 min (45). A review of the therapeutic potential of ayahuasca has suggested that there are a number of publications attesting to the potential benefits of ayahuasca and DMT in the areas of substance dependence, anxiety and depression with no serious adverse events reported (46).

Based on the data obtained from the Phase 1 study, a dose of 21.5 mg SPL026 given as a 2-part IV infusion over 10 min (6 mg/5 min and 15.5 mg/5 min) was selected as the dose to be taken into the Phase 2a study which assessed the efficacy, safety, tolerability and PD effects of SPL026 in participants with moderate-severe MDD (publication in preparation). This dose was selected as it satisfied all criteria regarding safety, tolerability, and psychedelic experience and, taking into account the opinion of the therapy team, was felt the most likely to be of therapeutic benefit in the target population of MDD patients. The rationale for this is that participants with MDD would be expected to have more psychological resistance to the subjective experience owing to their condition and may therefore be more likely to require a higher dose of SPL026. Additionally, the intense psychedelic state induced by 21.5 mg SPL026 may better help those participants overcome the cognitive rigidity typical of their condition (47–49) to allow the most therapeutic benefit.

There is a key limitation to this study, namely the small sample size. However, this was a Phase 1 study evaluating safety and tolerability of SPL026 in the first instance and as such the sample size was not powered to detect significant differences in PD scores between dosing groups and placebo. In addition, this was an exploratory evaluation of the tolerability of the psychedelic experience elicited by SPL026 in order to select a dose to progress into the Phase 2a study. The participants in this study were healthy, resilient and observed to be psychologically robust which may not necessarily be generalizable to the general population. Longer, more rigorous trials are needed to further explore long-term safety and tolerability of SPL026 not only in healthy subjects but also in relevant patient populations. Key strengths of the study were the rigorous and systematic testing of the safety and tolerability of different doses of SPL026 plus the very thorough evaluation of the psychological and PD effects of DMT in psychedelic-naïve subjects. This latter aspect of the study has highlighted differences in the psychometric profiles between SPL026 (DMT) and psilocybin which would be extremely useful to explore in future studies.

In conclusion, in healthy psychedelic-naïve participants, single doses of SPL026 (9, 12, 17 and 21.5 mg; administered by slow IV infusion) did not elicit any SAEs and did not produce any clinically relevant detrimental effects on a range of safety and tolerability parameters and on psychometric outcomes compared to placebo. Taken together, these findings support the exploration of 21.5 mg given over 10 min by IV infusion of SPL026 for the treatment of MDD including TRD, in a supervised setting with psychological support.

## Data availability statement

The original contributions presented in the study are included in the article/Supplementary material, further inquiries can be directed to the corresponding author.

## Ethics statement

The study involving humans were approved by Brent Research Ethics Committee. The study were conducted in accordance with the local legislation and institutional requirements. The participants provided their written informed consent to participate in this study.

## Author contributions

EJ: Conceptualization, Methodology, Project administration, Writing – original draft. DE: Conceptualization, Investigation, Supervision, Writing – review & editing. TBe: Conceptualization, Formal analysis, Writing – review & editing, Methodology, Writing – original draft. ZJ: Conceptualization, Writing – review & editing, Project administration. CT: Conceptualization, Writing – review & editing, Methodology. MG: Methodology, Writing – review & editing, Formal analysis, Project administration. CA: Formal analysis, Methodology, Writing – review & editing, Data curation. BW: Data curation, Formal analysis, Writing – review & editing, Methodology. TBa: Data curation, Writing – review & editing, Formal analysis, Methodology. GC: Writing – review & editing, Conceptualization, Investigation, Methodology. MJ: Conceptualization, Investigation, Methodology, Writing – review & editing. CH: Investigation, Writing – review & editing, Formal analysis. HT: Investigation, Methodology, Writing – review & editing. MB: Investigation, Writing – review & editing, Supervision. CR: Investigation, Supervision, Writing – review & editing, Methodology.
