# Supplementary material for: Safety, tolerability, pharmacodynamic and wellbeing effects of SPL026 (dimethyltryptamine fumarate) in healthy participants: a randomized, placebo-controlled phase 1 trial
Source: Front Psychiatry. 2024 Jan 11;14:1305796. doi: 10.3389/fpsyt.2023.1305796 (PMC10810248; doi:10.3389/fpsyt.2023.1305796)
Supplement: Supplementary file 1 [file Data_Sheet_1.docx]

**SUPPLEMENTARY INFORMATION**

**Assessments completed at screening and follow-up**

1. Electroencephalography (EEG) readings were taken from up to 90 min before until 4 hours after dosing; post-dose recording was paused for the post-dose integration session. Continuous pulse rate was collected by the EEG equipment for EEG methodological purposes only.
2. Outcome measure scales: Participants self-completed psychometric scales as follows.
   - At the screening visit (Visit 1): Warwick-Edinburgh Mental Wellbeing Scales (WEMWBS), Spielberger’s State-Trait Anxiety Inventory TRAIT subscale (STAI-T), Profile of Mood States (POMS), Brief Experiential Avoidance Questionnaire (BEAQ), Gratitude Questionnaire, Snaith Hamilton Anhedonia Pleasure Scale (SHAPS), Flourishing Scale (FS-8), Life Orientation Test (LOT), Meaning in Life Questionnaire (MLQ), Brief Resilience Scale (BRS), Dysfunctional Attitude Scale (DAS), Barrett Impulsivity Scale (BIS), Social Connectedness Scale (SCS), CompACT Scale, Openness Enriched Big Five Inventory (BFI), Metaphysical Beliefs Scale (MBS) and the Watts Connectedness Scale (WCS)
   - On Day 8: STAI-T, POMS, SHAPS, LOT, BIS, and DAS
   - On Day 15, and at 1 month (Day 30 ± 2 days) and 3 months (Day 90 ± 5 days) after dosing: WEMWBS, STAI-T, POMS, BEAQ, Gratitude Questionnaire, SHAPS, FS-8, LOT, MLQ, BRS, DAS, BIS, SCS, CompACT Scale, MBS, WCS, Psychological Insight Scale, and the PTCS. The 3-month assessment also included the Openness Enriched BFI.
3. Predictor measure scales: participants completed The PPS before dosing on Day 1.
4. Participant experience evaluation (acute effects) scales: Participants completed the following psychometric scales after dosing on Day 1: MEQ, EDI, EBI, CEQ, 5D-ASCQ, VAS, and the Metaphysical Experience Questionnaire (MPEQ).

**List of laboratory tests**

| ***Serum tests*** | |
| --- | --- |
| **Haematology:**   - Hemoglobin - Red blood cells - Mean corpuscular volume - Mean corpuscular hemoglobin - Mean corpuscular hemoglobin concentration - Hematocrit - White blood cells and differential (neutrophils, lymphocytes, monocytes, eosinophils, basophils) - Platelets - Reticulocytes   **Coagulation:**   - Activated partial thromboplastin time - Prothrombin time   **Serology (screening):**   - Hepatitis B surface antigen - Hepatitis C antibody - HIV screen (HIV 1 and 2)   **Female participants** **only (screening and Day -1):**   - Serum pregnancy test - Follicle stimulating hormone | **Clinical chemistry:**   - Urea - Uric acid - Creatinine - Creatinine phosphokinase - Total bilirubin - Total protein - Albumin - Globulin - Alkaline phosphatase - Aspartate transaminase - Alanine transaminase - Gamma-glutamyl transpeptidase - Glucose - Phosphate - Cholesterol - Triglycerides - Potassium - Sodium - Calcium - Chloride - Amylase |
| ***Urine tests*** | |
| **Urinalysis:**   - Dipstick: protein, blood, ketones, glucose, bilirubin, urobilinogen, leukocyte esterase, specific gravity, nitrites, pH - Microscopy: only if dipstick test for protein, blood, leukocyte esterase or nitrites was abnormal | **Other (screening and Day -1):**   - Drugs of abuse: amphetamines, cocaine, opiates, cannabis, barbiturates, and benzodiazepines - Cotinine (Phase 1 study only) - Alcohol |

These tests were done at screening, and on Day –1 and at 240 min after dosing on Day 1, unless otherwise stated in the table above. Additional safety assessments included physical examination, pulse rate, arterial blood pressure, tympanic temperature and 12-lead electrocardiogram (ECG). These were conducted at screening, and pre- and several times post-dose on Day 1, and Day 2, with the exception of 12-lead ECG, not done on Day 2.

**Table S1 Test statistics results of acute psychometric measures (other than VAS), and C_max_**

| **Dependent variable** | **Independent variable** | **df, N** | **Test** | **Statistics** | **p-value** | **p-value adjusted** |
| --- | --- | --- | --- | --- | --- | --- |
| MEQ | Group (placebo, SPL026 9, 12, 17, 21.5 mg) | 4, 30 | ANOVA | F = 5.324 | 0.003 | 0.016 |
|  | C_max_ | 20, 22 | Pearson | R = 0.27 | 0.221 | 0.348 |
|  | C_max_ x C_max_2 | 2/18, 22 | Quadratic regression | F = 11.46 | 0.001 | 0.006 |
|  | C_max_ (excluding 3 participants with C_max_ >80 ng/mL) | 17, 19 | Pearson | R = 0.71 | 0.001 | 0.006 |
| EDI | Group (placebo, SPL026 9, 12, 17, 21.5 mg) | 4, 30 | ANOVA | F = 3.969 | 0.013 | 0.038 |
|  | C_max_ | 20, 22 | Pearson | R = 0.4 | 0.067 | 0.127 |
|  | C_max_ x C_max_2 | 2/18, 22 | Quadratic regression | F = 7.2193 | 0.005 | 0.021 |
|  | C_max_ (excluding 3 participants with C_max_ >80 ng/mL) | 17, 19 | Pearson | R = 0.67 | 0.002 | 0.013 |
| EBI | Group (placebo, SPL026 9, 12, 17, 21.5 mg) | 4, 30 | ANOVA | F = 8.748 | <0.001 | 0.002 |
|  | C_max_ | 20, 22 | Pearson | R = 0.22 | 0.330 | 0.465 |
|  | C_max_ x C_max_2 | 2/18, 22 | Quadratic regression | F = 1.1433 | 0.341 | 0.475 |
|  | C_max_ (excluding 3 participants with C_max_ >80 ng/mL) | 17, 19 | Pearson | R = 0.3 | 0.234 | 0.365 |
| CEQ | Group (placebo, SPL026 9, 12, 17, 21.5 mg) | 4, 30 | ANOVA | F = 1.217 | 0.329 | 0.465 |
|  | C_max_ | 20, 22 | Pearson | R = 0.18 | 0.417 | 0.548 |
|  | C_max_ x C_max_2 | 2/18, 22 | Quadratic regression | F = 2.206 | 0.138 | 0.229 |
|  | C_max_ (excluding 3 participants with C_max_ >80 ng/mL) | 17, 19 | Pearson | R = 0.44 | 0.057 | 0.114 |
| 5D-ASCQ: Oceanic Boundlessness | Group (placebo, SPL026 9, 12, 17, 21.5 mg) | 4, 30 | ANOVA | F = 4.787 | 0.005 | 0.021 |
|  | C_max_ | 20, 22 | Pearson | R = 0.055 | 0.808 | 0.881 |
|  | C_max_ x C_max_2 | 2/18, 22 | Quadratic regression | F = 7.149 | 0.005 | 0.021 |
|  | C_max_ (excluding 3 participants with C_max_ >80 ng/mL) | 17, 19 | Pearson | R = 0.62 | 0.005 | 0.021 |
| 5D-ASCQ: Dread of Ego Dissolution | Group (placebo, SPL026 9, 12, 17, 21.5 mg) | 4, 30 | ANOVA | F = 2.539 | 0.065 | 0.126 |
|  | C_max_ | 20, 22 | Pearson | R = 0.19 | 0.403 | 0.544 |
|  | C_max_ x C_max_2 | 2/18, 22 | Quadratic regression | F = 4.028 | 0.035 | 0.081 |
|  | C_max_ (excluding 3 participants with C_max_ >80 ng/mL) | 17, 19 | Pearson | R = 0.53 | 0.019 | 0.054 |
| 5D-ASCQ: Visionary Restructuralization | Group (placebo, SPL026 9, 12, 17, 21.5 mg) | 4, 30 | ANOVA | F = 11.77 | < 0.001 | < 0.001 |
|  | C_max_ | 20, 22 | Pearson | R = 0.18 | 0.412 | 0.548 |
|  | Cmax x C_max_2 | 2/18, 22 | Quadratic regression | F = 6.475 | 0.007 | 0.024 |
|  | C_max_ (excluding 3 participants with C_max_ >80 ng/mL) | 17, 19 | Pearson | R = 0.61 | 0.006 | 0.021 |
| 5D-ASCQ: Auditory Alterations | Group (placebo, SPL026 9, 12, 17, 21.5 mg) | 4, 30 | ANOVA | F = 2.761 | 0.050 | 0.105 |
|  | C_max_ | 20, 22 | Pearson | R = 0.25 | 0.269 | 0.402 |
|  | Cmax x C_max_2 | 2/18, 22 | Quadratic regression | F = 2.43 | 0.115 | 0.201 |
|  | C_max_ (excluding 3 participants with C_max_ >80 ng/mL) | 17, 19 | Pearson | R = 0.48 | 0.036 | 0.081 |
| 5D-ASCQ: Vigilance Reduction | Group (placebo, SPL026 9, 12, 17, 21.5 mg) | 4, 30 | ANOVA | F = 1.407 | 0.261 | 0.396 |
|  | C_max_ | 20, 22 | Pearson | R = 0.062 | 0.783 | 0.860 |
|  | Cmax x C_max_2 | 2/18, 22 | Quadratic regression | F = 0.876 | 0.433 | 0.555 |
|  | C_max_ (excluding 3 participants with C_max_ >80 ng/mL) | 17, 19 | Pearson | R = 0.32 | 0.185 | 0.301 |
| C_max_ | Group (placebo, SPL026 9, 12, 17, 21.5 mg) | 3, 22 | ANOVA | F = 3.992 | 0.024 | 0.063 |

MEQ, Mystical Experience Questionnaire; C_max_, maximum plasma concentration; ANOVA, analysis of variance; EDI, Ego Dissolution Inventory; EBI, Emotional Breakthrough Inventory; CEQ, Challenging Experience Questionnaire; 5D-ASCQ, 5-Dimension Altered States of Consciousness Questionnaire.

**Table S2 Test statistics results of acute psychometric measure visual analog scales (VAS)**

| **Dependent Variable** | **Independent Variable** | **df, N** | **Test** | **Statistics** | **p-value** | **p-value adjusted** |
| --- | --- | --- | --- | --- | --- | --- |
| VAS drug intensity (Participant / Psychiatrist: IRVAS)* | Group (placebo, SPL026 9, 12, 17, 21.5 mg) | 4, 30 | ANOVA | F = 9.9733 |  |  |
|  | C_max_ | 20, 22 | Pearson | R = 0.15 |  |  |
|  | C_max_ x C_max_2 | 2/19, 22 | Quadratic regression | F = 8.192 |  |  |
|  | C_max_ (excluding 3 participants with C_max_ >80 ng/mL) | 17, 19 | Pearson | R = 0.61 |  |  |
| VAS drug Intensity (Participant: IRVAS) | Group (placebo, SPL026 9, 12, 17, 21.5 mg) | 4, 30 | ANOVA | F = 18.539 |  |  |
|  | C_max_ | 20, 22 | Pearson | R = 0.15 |  |  |
|  | C_max_ x C_max_2 | 2/19, 22 | Quadratic regression | F = 13.47 |  |  |
|  | C_max_ (excluding 3 participants with C_max_ >80 ng/mL) | 17, 19 | Pearson | R = 0.65 |  |  |
| VAS Altered sense of Space | Group (placebo, SPL026 9, 12, 17, 21.5 mg) | 4, 30 | ANOVA | F = 9.4798 |  |  |
|  | C_max_ | 20, 22 | Pearson | R = 0.17 |  |  |
|  | C_max_ x C_max_2 | 2/19, 22 | Quadratic regression | F = 8.344 |  |  |
|  | C_max_ (excluding 3 participants with C_max_ >80 ng/mL) | 17, 19 | Pearson | R = 0.6 |  |  |
| VAS Altered sense of Time | Group (placebo, SPL026 9, 12, 17, 21.5 mg) | 4, 30 | ANOVA | F = 6.020 |  |  |
|  | C_max_ | 20, 22 | Pearson | R = 0.069 |  |  |
|  | C_max_ x C_max_2 | 2/19, 22 | Quadratic regression | F = 7.06 |  |  |
|  | C_max_ (excluding 3 participants with C_max_ >80 ng/mL) | 17, 19 | Pearson | R = 0.53 |  |  |
| VAS Body energy | Group (placebo, SPL026 9, 12, 17, 21.5 mg) | 4, 30 | ANOVA | F = 4.628 |  |  |
|  | C_max_ | 20, 22 | Pearson | R = - 0.011 |  |  |
|  | C_max_ x C_max_2 | 2/19, 22 | Quadratic regression | F = 3.395 |  |  |
|  | C_max_ (excluding 3 participants with C_max_ >80 ng/mL) | 17, 19 | Pearson | R = 0.46 |  |  |
| VAS Buzzing sound | Group (placebo, SPL026 9, 12, 17, 21.5 mg) | 4, 30 | ANOVA | F = 2.096 |  |  |
|  | C_max_ | 20, 22 | Pearson | R = 0.1 |  |  |
|  | C_max_ x C_max_2 | 2/19, 22 | Quadratic regression | F = 0.3826 |  |  |
|  | C_max_ (excluding 3 participants with C_max_ >80 ng/mL) | 17, 19 | Pearson | R = 0.13 |  |  |
| VAS Challenging Experience | Group (placebo, SPL026 9, 12, 17, 21.5 mg) | 4, 30 | ANOVA | F = 5.683 |  |  |
|  | C_max_ | 20, 22 | Pearson | R = 0.44 |  |  |
|  | C_max_ x C_max_2 | 2/19, 22 | Quadratic regression | F = 3.228 |  |  |
|  | C_max_ (excluding 3 participants with C_max_ >80 ng/mL) | 17, 19 | Pearson | R = 0.41 |  |  |
| VAS Complex visual Images | Group (placebo, SPL026 9, 12, 17, 21.5 mg) | 4, 30 | ANOVA | F = 19.331 |  |  |
|  | C_max_ | 20, 22 | Pearson | R = 0.14 |  |  |
|  | C_max_ x C_max_2 | 2/19, 22 | Quadratic regression | F = 1.775 |  |  |
|  | C_max_ (excluding 3 participants with C_max_ >80 ng/mL) | 17, 19 | Pearson | R = 0.38 |  |  |
| VAS Displaced from the body | Group (placebo, SPL026 9, 12, 17, 21.5 mg) | 4, 30 | ANOVA | F = 4.8264 |  |  |
|  | C_max_ | 20, 22 | Pearson | R = 0.0023 |  |  |
|  | C_max_ x C_max_2 | 2/19, 22 | Quadratic regression | F = 7.707 |  |  |
|  | C_max_ (excluding 3 participants with C_max_ >80 ng/mL) | 17, 19 | Pearson | R = 0.61 |  |  |
| VAS Dreamlike quality | Group (placebo, SPL026 9, 12, 17, 21.5 mg) | 4, 30 | ANOVA | F = 4.4861 |  |  |
|  | C_max_ | 20, 22 | Pearson | R = 0.16 |  |  |
|  | C_max_ x C_max_2 | 2/19, 22 | Quadratic regression | F = 3.878 |  |  |
|  | C_max_ (excluding 3 participants with C_max_ >80 ng/mL) | 17, 19 | Pearson | R = 0.51 |  |  |
| VAS Ego dissolution | Group (placebo, SPL026 9, 12, 17, 21.5 mg) | 4, 30 | ANOVA | F = 3.9501 |  |  |
|  | C_max_ | 20, 22 | Pearson | R = 0.21 |  |  |
|  | C_max_ x C_max_2 | 2/19, 22 | Quadratic regression | F = 4.348 |  |  |
|  | C_max_ (excluding 3 participants with C_max_ >80 ng/mL) | 17, 19 | Pearson | R = 0.56 |  |  |
| VAS Forgot it was an experiment | Group (placebo, SPL026 9, 12, 17, 21.5 mg) | 4, 30 | ANOVA | F = 5.6636 |  |  |
|  | C_max_ | 20, 22 | Pearson | R = 0.29 |  |  |
|  | C_max_ x C_max_2 | 2/19, 22 | Quadratic regression | F = 3.423 |  |  |
|  | C_max_ (excluding 3 participants with C_max_ >80 ng/mL) | 17, 19 | Pearson | R = 0.49 |  |  |
| VAS Geometric patterns | Group (placebo, SPL026 9, 12, 17, 21.5 mg) | 4, 30 | ANOVA | F = 25.595 |  |  |
|  | C_max_ | 20, 22 | Pearson | R = 0.071 |  |  |
|  | C_max_ x C_max_2 | 2/19, 22 | Quadratic regression | F = 0.9089 |  |  |
|  | C_max_ (excluding 3 participants with C_max_ >80 ng/mL) | 17, 19 | Pearson | R = 0.24 |  |  |
| VAS Immerison in different dimension | Group (placebo, SPL026 9, 12, 17, 21.5 mg) | 4, 30 | ANOVA | F = 4.617 |  |  |
|  | C_max_ | 20, 22 | Pearson | R = 0.11 |  |  |
|  | C_max_ x C_max_2 | 2/19, 22 | Quadratic regression | F = 9.043 |  |  |
|  | C_max_ (excluding 3 participants with C_max_ >80 ng/mL) | 17, 19 | Pearson | R = 0.66 |  |  |
| VAS Insight on life | Group (placebo, SPL026 9, 12, 17, 21.5 mg) | 4, 30 | ANOVA | F = 10.243 |  |  |
|  | C_max_ | 20, 22 | Pearson | R = 0.22 |  |  |
|  | C_max_ x C_max_2 | 2/19, 22 | Quadratic regression | F = 1.366 |  |  |
|  | C_max_ (excluding 3 participants with C_max_ >80 ng/mL) | 17, 19 | Pearson | R = 0.33 |  |  |
| VAS It felt normal | Group (placebo, SPL026 9, 12, 17, 21.5 mg) | 4, 30 | ANOVA | F = 5.1141 |  |  |
|  | C_max_ | 20, 22 | Pearson | R = -0.017 |  |  |
|  | C_max_ x C_max_2 | 2/19, 22 | Quadratic regression | F = 0.3445 |  |  |
|  | C_max_ (excluding 3 participants with C_max_ >80 ng/mL) | 17, 19 | Pearson | R = -0.19 |  |  |
| VAS Meaningful experience | Group (placebo, SPL026 9, 12, 17, 21.5 mg) | 4, 30 | ANOVA | F = 6.6032 |  |  |
|  | C_max_ | 20, 22 | Pearson | R = 0.0077 |  |  |
|  | C_max_ x C_max_2 | 2/19, 22 | Quadratic regression | F = 2.604 |  |  |
|  | C_max_ (excluding 3 participants with C_max_ >80 ng/mL) | 17, 19 | Pearson | R = 0.36 |  |  |
| VAS More real than reality | Group (placebo, SPL026 9, 12, 17, 21.5 mg) | 4, 30 | ANOVA | F = 2.6327 |  |  |
|  | C_max_ | 20, 22 | Pearson | R = 0.15 |  |  |
|  | C_max_ x C_max_2 | 2/19, 22 | Quadratic regression | F = 4.212 |  |  |
|  | C_max_ (excluding 3 participants with C_max_ >80 ng/mL) | 17, 19 | Pearson | R = 0.53 |  |  |
| VAS Open to all emotions | Group (placebo, SPL026 9, 12, 17, 21.5 mg) | 4, 30 | ANOVA | F = 10.243 |  |  |
|  | C_max_ | 20, 22 | Pearson | R = 0.13 |  |  |
|  | C_max_ x C_max_2 | 2/19, 22 | Quadratic regression | F = 1.179 |  |  |
|  | C_max_ (excluding 3 participants with C_max_ >80 ng/mL) | 17, 19 | Pearson | R = 0.21 |  |  |
| VAS Pleasurable experience | Group (placebo, SPL026 9, 12, 17, 21.5 mg) | 4, 30 | ANOVA | F = 10.243 |  |  |
|  | C_max_ | 20, 22 | Pearson | R = -0.029 |  |  |
|  | C_max_ x C_max_2 | 2/19, 22 | Quadratic regression | F = 1.437 |  |  |
|  | C_max_ (excluding 3 participants with C_max_ >80 ng/mL) | 17, 19 | Pearson | R = 0.35 |  |  |
| VAS Presence of beings | Group (placebo, SPL026 9, 12, 17, 21.5 mg) | 4, 30 | ANOVA | F = 2.1144 |  |  |
|  | C_max_ | 20, 22 | Pearson | R = 0.19 |  |  |
|  | C_max_ x C_max_2 | 2/19, 22 | Quadratic regression | F = 4.338 |  |  |
|  | C_max_ (excluding 3 participants with C_max_ >80 ng/mL) | 17, 19 | Pearson | R = 0.61 |  |  |
| VAS Richness of experience | Group (placebo, SPL026 9, 12, 17, 21.5 mg) | 4, 30 | ANOVA | F = 11.248 |  |  |
|  | C_max_ | 20, 22 | Pearson | R = 0.025 |  |  |
|  | C_max_ x C_max_2 | 2/19, 22 | Quadratic regression | F = 9.325 |  |  |
|  | C_max_ (excluding 3 participants with C_max_ >80 ng/mL) | 17, 19 | Pearson | R = 0.55 |  |  |
| VAS Sense of gratitude | Group (placebo, SPL026 9, 12, 17, 21.5 mg) | 4, 30 | ANOVA | F = 1.9753 |  |  |
|  | C_max_ | 20, 22 | Pearson | R = -0.099 |  |  |
|  | C_max_ x C_max_2 | 2/19, 22 | Quadratic regression | F = 5.684 |  |  |
|  | C_max_ (excluding 3 participants with C_max_ >80 ng/mL) | 17, 19 | Pearson | R = 0.47 |  |  |
| VAS Sounds influence visual | Group (placebo, SPL026 9, 12, 17, 21.5 mg) | 4, 30 | ANOVA | F = 9.2606 |  |  |
|  | C_max_ | 20, 22 | Pearson | R = 0.028 |  |  |
|  | C_max_ x C_max_2 | 2/19, 22 | Quadratic regression | F = 0.5842 |  |  |
|  | C_max_ (excluding 3 participants with C_max_ >80 ng/mL) | 17, 19 | Pearson | R = 0.19 |  |  |
| VAS Unusual bodily sensations | Group (placebo, SPL026 9, 12, 17, 21.5 mg) | 4, 30 | ANOVA | F = 6.8984 |  |  |
|  | C_max_ | 20, 22 | Pearson | R = -0.027 |  |  |
|  | C_max_ x C_max_2 | 2/19, 22 | Quadratic regression | F = 1.481 |  |  |
|  | C_max_ (excluding 3 participants with C_max_ >80 ng/mL) | 17, 19 | Pearson | R = 0.19 |  |  |
| VAS Vibrating body | Group (placebo, SPL026 9, 12, 17, 21.5 mg) | 4, 30 | ANOVA | F = 3.4347 |  |  |
|  | C_max_ | 20, 22 | Pearson | R = -0.0029 |  |  |
|  | C_max_ x C_max_2 | 2/19, 22 | Quadratic regression | F = 0.09426 |  |  |
|  | C_max_ (excluding 3 participants with C_max_ >80 ng/mL) | 17, 19 | Pearson | R = 0.0079 |  |  |
| VAS Visual immersion | Group (placebo, SPL026 9, 12, 17, 21.5 mg) | 4, 30 | ANOVA | F = 13.743 |  |  |
|  | C_max_ | 20, 22 | Pearson | R = 0.053 |  |  |
|  | C_max_ x C_max_2 | 2/19, 22 | Quadratic regression | F = 4.186 |  |  |
|  | C_max_ (excluding 3 participants with C_max_ >80 ng/mL) | 17, 19 | Pearson | R = 0.49 |  |  |

*Question asked for IRVAS: “How intense was the experience?”. Both participant and psychiatrist/therapist answered independently. Results averaged for Participant / Psychiatrist analysis.

VAS, visual analog scale; df, degrees of freedom; ANOVA, analysis of variance; IRVAS, IRVAS, Intensity Rating Visual Analogue Scale; C_max_, maximum plasma concentration.

**Table S3 Summary of Warwick-Edinburgh Mental Wellbeing Scale after single IV doses of 9–‍21.5 mg SPL026 in healthy participants (PD population)**

| **Treatment** | **Planned relative time** | **Mean (SD)** | **Mean change from baseline (SD)** |
| --- | --- | --- | --- |
| Placebo (N=8) | Screening | 62.9 (6.51) | - |
|  | Day 15 | 59.4 (5.55) | –3.5 (5.81) |
|  | Day 30 | 61.6 (4.50) | –1.3 (7.19) |
|  | Day 90 | 61.3 (5.01 | –1.6 (7.50) |
| SPL026 9 mg (N=5)* | Screening | 57.2 (6.83) | - |
|  | Day 15^†^ | 55.3 (12.92) | –2.3 (5.12) |
|  | Day 30^†^ | 59.3 (7.41) | –1.8 (2.99) |
|  | Day 90^†^ | 56.8 (10.53) | –0.8 (2.87) |
| SPL026 12 mg (N=6) | Screening | 63.7 (5.39) | - |
|  | Day 15 | 60.0 (8.15) | –3.7 (3.98) |
|  | Day 30 | 57.3 (11.79) | –6.3 (8.12) |
|  | Day 90 | 59.0 (9.55) | –4.7 (4.37) |
| SPL026 17 mg (N=5)* | Screening | 61.6 (7.50) | - |
|  | Day 15 | 60.2 (8.87) | –1.4 (4.04) |
|  | Day 30 | 62.8 (8.64) | 1.2 (8.76) |
|  | Day 90 | 60.6 (6.54) | –1.0 (3.74) |
| SPL026 21.5 mg (N=6) | Screening | 60.2 (8.64) | - |
|  | Day 15 | 58.5 (9.61) | –1.7 (2.07) |
|  | Day 30 | 56.0 (8.07) | –4.2 (6.11) |
|  | Day 90 | 56.9 (7.47) | –3.2 (7.22) |

*Participants 1006 (9 mg SPL026) and 3001 (17 mg SPL026) were excluded from the PK concentration and parameter populations. ^†^N=4.

IV, intravenous; PD, pharmacodynamic; SD, standard deviation; PK, pharmacokinetics.

**Table S4 Summary of Spielberger’s State-Trait Anxiety Inventory - TRAIT subscale** **after single IV doses of 9–‍21.5 mg SPL026 in healthy participants (PD population)**

| **Treatment** | **Planned relative time** | **Mean (SD)** | **Mean change from baseline (SD)** |
| --- | --- | --- | --- |
| Placebo (N=8) | Screening | 24.6 (5.55) | 1.3 (5.41) |
|  | Day 15 | 24.7 (4.11) | 3.1 (5.41) |
|  | Day 30 | 27.8 (6.16) | 1.1 (2.64) |
|  | Day 90 | 25.8 (4.74) | 1.3 (4.56) |
| SPL026 9 mg (N=5)* | Screening | 30.4 (9.21) | 3.6 (5.13) |
|  | Day 15^†^ | 34.0 (13.25) | 2.8 (4.43) |
|  | Day 30^†^ | 34.3 (14.1) | –0.3 (2.63) |
|  | Day 90^†^ | 31.3 (11.93) | 1.3 (2.87) |
| SPL026 12 mg (N=6) | Screening | 25.3 (7.17) | 0.8 (3.06) |
|  | Day 15 | 26.2 (6.24) | 3.2 (6.52) |
|  | Day 30 | 28.5 (8.55) | 4 (5.9) |
|  | Day 90 | 29.3 (8.64) | 3.0 (2.97) |
| SPL026 17 mg (N=5)* | Screening | 29.8 (5.4) | 1.2 (4.71) |
|  | Day 15 | 31.0 (8.25) | –1.4 (3.65) |
|  | Day 30 | 28.4 (8.26) | –0.8 (3.9) |
|  | Day 90 | 29.0 (7.65) | 1.8 (7.56) |
| SPL026 21.5 mg (N=6) | Screening | 27.3 (7.37) | 2.7 (3.27) |
|  | Day 15 | 30.0 (7.10) | 2.2 (2.64) |
|  | Day 30 | 29.5 (8.31) | 3.2 (4.84) |
|  | Day 90 | 30.6 (8.87) | 3.2 (5.04) |

*Participants 1006 (9 mg SPL026) and 3001 (17 mg SPL026) were excluded from the PK concentration and parameter populations. ^†^N=4.

IV, intravenous; PD, pharmacodynamic; SD, standard deviation; PK, pharmacokinetics.

**Table S5 Test statistics results of long-term psychometric measures**

| **Dependent Variable** | **Independent variable** | **df, N** | **Test** | **Statistics** | **p-value** | **p-value adjusted** |
| --- | --- | --- | --- | --- | --- | --- |
| POMS | Group (placebo, SPL026 9, 12, 17, 21.5 mg) | 16, 30 | LMR: Score ~ Group*factor(Day)+(1\| ID) | Χ^2^ = 20.9917 | 0.179 | 0.617 |
|  | C_max_ | 4, 22 | LMR: Score ~ scale(C_max_)*factor(Day)+(1\| ID) | Χ^2^ = 1.0807 | 0.897 | 0.942 |
|  | C_max_ x C_max_2 | 8, 22 | LMR: Score ~ poly(scale(C_max_), 2,raw=T)*factor(Day)+(1\| ID) | Χ^2^ = 9.6075 | 0.294 | 0.633 |
|  | C_max_ (excluding 3 participants with C_max_ >80 ng/mL) | 4, 19 | LMR: Score ~ scale(C_max_)*factor(Day)+(1\| ID) | Χ^2^ = 4.6105 | 0.330 | 0.633 |
| BEAQ | Group (placebo, SPL026 9, 12, 17, 21.5 mg) | 12, 30 | LMR: Score ~ Group*factor(Day)+(1\| ID) | Χ^2^ = 11.228 | 0.509 | 0.737 |
|  | C_max_ | 3, 22 | LMR: Score ~ scale(C_max_)*factor(Day)+(1\| ID) | Χ^2^ = 2.6055 | 0.457 | 0.737 |
|  | C_max_ x C_max_2 | 6, 22 | LMR: Score ~ poly(scale(C_max_), 2,raw=T)*factor(Day)+(1\| ID) | Χ^2^ = 3.8821 | 0.693 | 0.819 |
|  | C_max_ (excluding 3 participants with C_max_ >80 ng/mL) | 3, 19 | LMR: Score ~ scale(C_max_)*factor(Day)+(1\| ID) | Χ^2^ = 1.7239 | 0.632 | 0.783 |
| GQ-6 | Group (placebo, SPL026 9, 12, 17, 21.5 mg) | 12, 30 | LMR: Score ~ Group*factor(Day)+(1\| ID) | Χ^2^ = 11.7238 | 0.468 | 0.737 |
|  | C_max_ | 3, 22 | LMR: Score ~ scale(C_max_)*factor(Day)+(1\| ID) | Χ^2^ = 2.7584 | 0.430 | 0.731 |
|  | C_max_ x C_max_2 | 6, 22 | LMR: Score ~ poly(scale(C_max_), 2,raw=T)*factor(Day)+(1\| ID) | Χ^2^ = 9.4197 | 0.151 | 0.578 |
|  | C_max_ (excluding 3 participants with C_max_ >80 ng/mL) | 3, 19 | LMR: Score ~ scale(C_max_)*factor(Day)+(1\| ID) | Χ^2^ = 6.2305 | 0.101 | 0.519 |
| SHAPS | Group (placebo, SPL026 9, 12, 17, 21.5 mg) | 16, 30 | LMR: Score ~ Group*factor(Day)+(1\| ID) | Χ^2^ = 12.3018 | 0.723 | 0.825 |
|  | C_max_ | 4, 22 | LMR: Score ~ scale(C_max_)*factor(Day)+(1\| ID) | Χ^2^ = 1.9046 | 0.753 | 0.844 |
|  | C_max_ x C_max_2 | 8, 22 | LMR: Score ~ poly(scale(C_max_), 2,raw=T)*factor(Day)+(1\| ID) | Χ^2^ = 2.7088 | 0.951 | 0.972 |
|  | C_max_ (excluding 3 participants with C_max_ >80 ng/mL) | 4, 19 | LMR: Score ~ scale(C_max_)*factor(Day)+(1\| ID) | Χ^2^ = 0.6241 | 0.960 | 0.972 |
| FS-8 | Group (placebo, SPL026 9, 12, 17, 21.5 mg) | 12, 30 | LMR: Score ~ Group*factor(Day)+(1\| ID) | Χ^2^ = 10.8920 | 0.538 | 0.753 |
|  | C_max_ | 3, 22 | LMR: Score ~ scale(C_max_)*factor(Day)+(1\| ID) | Χ^2^ = 5.3785 | 0.146 | 0.578 |
|  | C_max_ x C_max_2 | 6, 22 | LMR: Score ~ poly(scale(C_max_), 2,raw=T)*factor(Day)+(1\| ID) | Χ^2^ = 10.1771 | 0.117 | 0.519 |
|  | C_max_ (excluding 3 participants with C_max_ >80 ng/mL) | 3, 19 | LMR: Score ~ scale(C_max_)*factor(Day)+(1\| ID) | Χ^2^ = 1.7147 | 0.634 | 0.783 |
| LOT | Group (placebo, SPL026 9, 12, 17, 21.5 mg) | 16, 30 | LMR: Score ~ Group*factor(Day)+(1\| ID) | Χ^2^ = 18.2452 | 0.310 | 0.633 |
|  | C_max_ | 4, 22 | LMR: Score ~ scale(C_max_)*factor(Day)+(1\| ID) | Χ^2^ = 6.9587 | 0.138 | 0.578 |
|  | C_max_ x C_max_2 | 8, 22 | LMR: Score ~ poly(scale(C_max_), 2,raw=T)*factor(Day)+(1\| ID) | Χ^2^ = 13.9050 | 0.084 | 0.519 |
|  | C_max_ (excluding 3 participants with C_max_ >80 ng/mL) | 4, 19 | LMR: Score ~ scale(C_max_)*factor(Day)+(1\| ID) | Χ^2^ = 8.8587 | 0.065 | 0.519 |
| MLQ: Presence of meaning | Group (placebo, SPL026 9, 12, 17, 21.5 mg) | 12, 30 | LMR: Score ~ Group*factor(Day)+(1\| ID) | Χ^2^ = 10.1129 | 0.606 | 0.783 |
|  | C_max_ | 3, 22 | LMR: Score ~ scale(C_max_)*factor(Day)+(1\| ID) | Χ^2^ = 7.5653 | 0.056 | 0.519 |
|  | C_max_ x C_max_2 | 6, 22 | LMR: Score ~ poly(scale(C_max_), 2,raw=T)*factor(Day)+(1\| ID) | Χ^2^ = 14.4745 | 0.025 | 0.416 |
|  | C_max_ (excluding 3 participants with C_max_ >80 ng/mL) | 3, 19 | LMR: Score ~ scale(C_max_)*factor(Day)+(1\| ID) | Χ^2^ = 3.3194 | 0.345 | 0.644 |
| MLQ: Search for meaning | Group (placebo, SPL026 9, 12, 17, 21.5 mg) | 12, 30 | LMR: Score ~ Group*factor(Day)+(1\| ID) | Χ^2^ = 15.5450 | 0.213 | 0.617 |
|  | C_max_ | 3, 22 | LMR: Score ~ scale(C_max_)*factor(Day)+(1\| ID) | Χ^2^ = 0.9973 | 0.802 | 0.879 |
|  | C_max_ x C_max_2 | 6, 22 | LMR: Score ~ poly(scale(C_max_), 2,raw=T)*factor(Day)+(1\| ID) | Χ^2^ = 3.6288 | 0.727 | 0.825 |
|  | C_max_ (excluding 3 participants with C_max_ >80 ng/mL) | 3, 19 | LMR: Score ~ scale(C_max_)*factor(Day)+(1\| ID) | Χ^2^ = 2.3842 | 0.497 | 0.737 |
| BRS | Group (placebo, SPL026 9, 12, 17, 21.5 mg) | 12, 30 | LMR: Score ~ Group*factor(Day)+(1\| ID) | Χ^2^ = 13.9994 | 0.301 | 0.633 |
|  | C_max_ | 3, 22 | LMR: Score ~ scale(C_max_)*factor(Day)+(1\| ID) | Χ^2^ = 1.3428 | 0.719 | 0.825 |
|  | C_max_ x C_max_2 | 6, 22 | LMR: Score ~ poly(scale(C_max_), 2,raw=T)*factor(Day)+(1\| ID) | Χ^2^ = 7.7211 | 0.259 | 0.633 |
|  | C_max_ (excluding 3 participants with C_max_ >80 ng/mL) | 3, 19 | LMR: Score ~ scale(C_max_)*factor(Day)+(1\| ID) | Χ^2^ = 7.7566 | 0.051 | 0.519 |
| DAS | Group (placebo, SPL026 9, 12, 17, 21.5 mg) | 16, 30 | LMR: Score ~ Group*factor(Day)+(1\| ID) | Χ^2^ = 12.8389 | 0.685 | 0.819 |
|  | C_max_ | 4, 22 | LMR: Score ~ scale(C_max_)*factor(Day)+(1\| ID) | Χ^2^ = 5.0991 | 0.277 | 0.633 |
|  | C_max_ x C_max_2 | 8, 22 | LMR: Score ~ poly(scale(C_max_), 2,raw=T)*factor(Day)+(1\| ID) | Χ^2^ = 7.4780 | 0.486 | 0.737 |
|  | C_max_ (excluding 3 participants with C_max_ >80 ng/mL) | 4, 19 | LMR: Score ~ scale(C_max_)*factor(Day)+(1\| ID) | Χ^2^ = 5.2090 | 0.267 | 0.633 |
| BIS | Group (placebo, SPL026 9, 12, 17, 21.5 mg) | 16, 30 | LMR: Score ~ Group*factor(Day)+(1\| ID) | Χ^2^ = 7.5620 | 0.961 | 0.972 |
|  | C_max_ | 4, 22 | LMR: Score ~ scale(C_max_)*factor(Day)+(1\| ID) | Χ^2^ = 4.8442 | 0.304 | 0.633 |
|  | C_max_ x C_max_2 | 8, 22 | LMR: Score ~ poly(scale(C_max_), 2,raw=T)*factor(Day)+(1\| ID) | Χ^2^ = 7.2187 | 0.513 | 0.737 |
|  | C_max_ (excluding 3 participants with C_max_ >80 ng/mL) | 4, 19 | LMR: Score ~ scale(C_max_)*factor(Day)+(1\| ID) | Χ^2^ = 0.3920 | 0.983 | 0.983 |
| SCS | Group (placebo, SPL026 9, 12, 17, 21.5 mg) | 12, 30 | LMR: Score ~ Group*factor(Day)+(1\| ID) | Χ^2^ = 16.7752 | 0.158 | 0.578 |
|  | C_max_ | 3, 22 | LMR: Score ~ scale(C_max_)*factor(Day)+(1\| ID) | Χ^2^ = 7.2795 | 0.064 | 0.519 |
|  | C_max_ x C_max_2 | 6, 22 | LMR: Score ~ poly(scale(C_max_), 2,raw=T)*factor(Day)+(1\| ID) | Χ^2^ = 7.5111 | 0.276 | 0.633 |
|  | C_max_ (excluding 3 participants with C_max_ >80 ng/mL) | 3, 19 | LMR: Score ~ scale(C_max_)*factor(Day)+(1\| ID) | Χ^2^ = 2.2747 | 0.517 | 0.737 |
| CompACT | Group (placebo, SPL026 9, 12, 17, 21.5 mg) | 12, 30 | LMR: Score ~ Group*factor(Day)+(1\| ID) | Χ^2^ = 9.9687 | 0.619 | 0.783 |
|  | C_max_ | 3, 22 | LMR: Score ~ scale(C_max_)*factor(Day)+(1\| ID) | Χ^2^ = 4.7161 | 0.194 | 0.617 |
|  | C_max_ x C_max_2 | 6, 22 | LMR: Score ~ poly(scale(C_max_), 2,raw=T)*factor(Day)+(1\| ID) | Χ^2^ = 10.7516 | 0.096 | 0.519 |
|  | C_max_ (excluding 3 participants with C_max_ >80 ng/mL) | 3, 19 | LMR: Score ~ scale(C_max_)*factor(Day)+(1\| ID) | Χ^2^ = 4.3132 | 0.230 | 0.622 |
| BFI: Agreeableness | Group (placebo, SPL026 9, 12, 17, 21.5 mg) | 4, 30 | LMR: Score ~ Group*factor(Day)+(1\| ID) | Χ^2^ = 26.5357 | < 0.001 | 0.002 |
|  | C_max_ | 1, 22 | LMR: Score ~ scale(C_max_)*factor(Day)+(1\| ID) | Χ^2^ = 12.7669 | < 0.001 | 0.015 |
|  | C_max_ x C_max_2 | 2, 22 | LMR: Score ~ poly(scale(C_max_), 2,raw=T)*factor(Day)+(1\| ID) | Χ^2^ = 14.0632 | 0.001 | 0.025 |
|  | C_max_ (excluding 3 participants with C_max_ >80 ng/mL) | 1, 19 | LMR: Score ~ scale(C_max_)*factor(Day)+(1\| ID) | Χ^2^ = 6.5891 | 0.010 | 0.215 |
| BFI: Conscientiousness | Group (placebo, SPL026 9, 12, 17, 21.5 mg) | 4, 30 | LMR: Score ~ Group*factor(Day)+(1\| ID) | Χ^2^ = 3.7294 | 0.444 | 0.731 |
|  | C_max_ | 1, 22 | LMR: Score ~ scale(C_max_)*factor(Day)+(1\| ID) | Χ^2^ = 2.5921 | 0.107 | 0.519 |
|  | C_max_ x C_max_2 | 2, 22 | LMR: Score ~ poly(scale(C_max_), 2,raw=T)*factor(Day)+(1\| ID) | Χ^2^ = 4.3445 | 0.114 | 0.519 |
|  | C_max_ (excluding 3 participants with C_max_ >80 ng/mL) | 1, 19 | LMR: Score ~ scale(C_max_)*factor(Day)+(1\| ID) | Χ^2^ = 0.5110 | 0.475 | 0.737 |
| BFI: Extraversion | Group (placebo, SPL026 9, 12, 17, 21.5 mg) | 4, 30 | LMR: Score ~ Group*factor(Day)+(1\| ID) | Χ^2^ = 5.2621 | 0.261 | 0.633 |
|  | C_max_ | 1, 22 | LMR: Score ~ scale(C_max_)*factor(Day)+(1\| ID) | Χ^2^ = 1.5928 | 0.207 | 0.617 |
|  | C_max_ x C_max_2 | 2, 22 | LMR: Score ~ poly(scale(C_max_), 2,raw=T)*factor(Day)+(1\| ID) | Χ^2^ = 2.2666 | 0.322 | 0.633 |
|  | C_max_ (excluding 3 participants with C_max_ >80 ng/mL) | 1, 19 | LMR: Score ~ scale(C_max_)*factor(Day)+(1\| ID) | Χ^2^ = 2.7166 | 0.099 | 0.519 |
| BFI: Neuroticism | Group (placebo, SPL026 9, 12, 17, 21.5 mg) | 4, 30 | LMR: Score ~ Group*factor(Day)+(1\| ID) | Χ^2^ = 1.6157 | 0.806 | 0.879 |
|  | C_max_ | 1, 22 | LMR: Score ~ scale(C_max_)*factor(Day)+(1\| ID) | Χ^2^ = 0.1612 | 0.688 | 0.819 |
|  | C_max_ x C_max_2 | 2, 22 | LMR: Score ~ poly(scale(C_max_), 2,raw=T)*factor(Day)+(1\| ID) | Χ^2^ = 1.6595 | 0.436 | 0.731 |
|  | C_max_ (excluding 3 participants with C_max_ >80 ng/mL) | 1, 19 | LMR: Score ~ scale(C_max_)*factor(Day)+(1\| ID) | Χ^2^ = 0.3168 | 0.574 | 0.761 |
| BFI: Openness | Group (placebo, SPL026 9, 12, 17, 21.5 mg) | 4, 30 | LMR: Score ~ Group*factor(Day)+(1\| ID) | Χ^2^ = 3.9209 | 0.417 | 0.731 |
|  | C_max_ | 1, 22 | LMR: Score ~ scale(C_max_)*factor(Day)+(1\| ID) | Χ^2^ = 0.5888 | 0.443 | 0.731 |
|  | C_max_ x C_max_2 | 2, 22 | LMR: Score ~ poly(scale(C_max_), 2,raw=T)*factor(Day)+(1\| ID) | Χ^2^ = 1.8900 | 0.389 | 0.710 |
|  | C_max_ (excluding 3 participants with C_max_ >80 ng/mL) | 1, 19 | LMR: Score ~ scale(C_max_)*factor(Day)+(1\| ID) | Χ^2^ = 0.3071 | 0.580 | 0.761 |
| PIS 1-6 | Group (placebo, SPL026 9, 12, 17, 21.5 mg) | 8, 30 | LMR: Score ~ Group*factor(Day)+(1\| ID) | Χ^2^ = 3.8910 | 0.867 | 0.922 |
|  | C_max_ | 2, 22 | LMR: Score ~ scale(C_max_)*factor(Day)+(1\| ID) | Χ^2^ = 4.3691 | 0.113 | 0.519 |
|  | C_max_ x C_max_2 | 4, 22 | LMR: Score ~ poly(scale(C_max_), 2,raw=T)*factor(Day)+(1\| ID) | Χ^2^ = 4.5946 | 0.332 | 0.633 |
|  | C_max_ (excluding 3 participants with C_max_ >80 ng/mL) | 2, 19 | LMR: Score ~ scale(C_max_)*factor(Day)+(1\| ID) | Χ^2^ = 0.3013 | 0.860 | 0.922 |
| PIS 7 | Group (placebo, SPL026 9, 12, 17, 21.5 mg) | 8, 30 | LMR: Score ~ Group*factor(Day)+(1\| ID) | Χ^2^ = 9.5835 | 0.295 | 0.633 |
|  | C_max_ | 2, 22 | LMR: Score ~ scale(C_max_)*factor(Day)+(1\| ID) | Χ^2^ = 2.9937 | 0.224 | 0.622 |
|  | C_max_ x C_max_2 | 4, 22 | LMR: Score ~ poly(scale(C_max_), 2,raw=T)*factor(Day)+(1\| ID) | Χ^2^ = 5.9126 | 0.206 | 0.617 |
|  | C_max_ (excluding 3 participants with C_max_ >80 ng/mL) | 2, 19 | LMR: Score ~ scale(C_max_)*factor(Day)+(1\| ID) | Χ^2^ = 3.2335 | 0.199 | 0.617 |
| WCS | Group (placebo, SPL026 9, 12, 17, 21.5 mg) | 12, 30 | LMR: Score ~ Group*factor(Day)+(1\| ID) | Χ^2^ = 10.4280 | 0.579 | 0.761 |
|  | C_max_ | 3, 22 | LMR: Score ~ scale(C_max_)*factor(Day)+(1\| ID) | Χ^2^ = 7.1043 | 0.069 | 0.519 |
|  | C_max_ x C_max_2 | 6, 22 | LMR: Score ~ poly(scale(C_max_), 2,raw=T)*factor(Day)+(1\| ID) | Χ^2^ = 13.9444 | 0.030 | 0.424 |
|  | C_max_ (excluding 3 participants with C_max_ >80 ng/mL) | 3, 19 | LMR: Score ~ scale(C_max_)*factor(Day)+(1\| ID) | Χ^2^ = 1.9659 | 0.580 | 0.761 |

POMS, Profile of Mood States; BEAQ, Brief Experiential Avoidance Questionnaire; GC-6, Gratitude Questionnaire; SHAPS, Snaith Hamilton Anhedonia Pleasure Scale; FS-8, Flourishing Scale; LOT, Life Orientation Test; MLQ, Meaning in Life Questionnaire; BRS, Brief Resilience Scale; DAS, Dysfunctional Attitude Scale; BIS, Barrett Impulsivity Scale; SCS, Social Connectedness Scale; CompACT Scale; BFI, Openness Enriched Big Five Inventory; PIS, Psychological Insight Scale; WCS, Watts Connectedness Scale.

**Figure S1 5D-ASCQ as 5 dimensions and as 11 subscales**

**
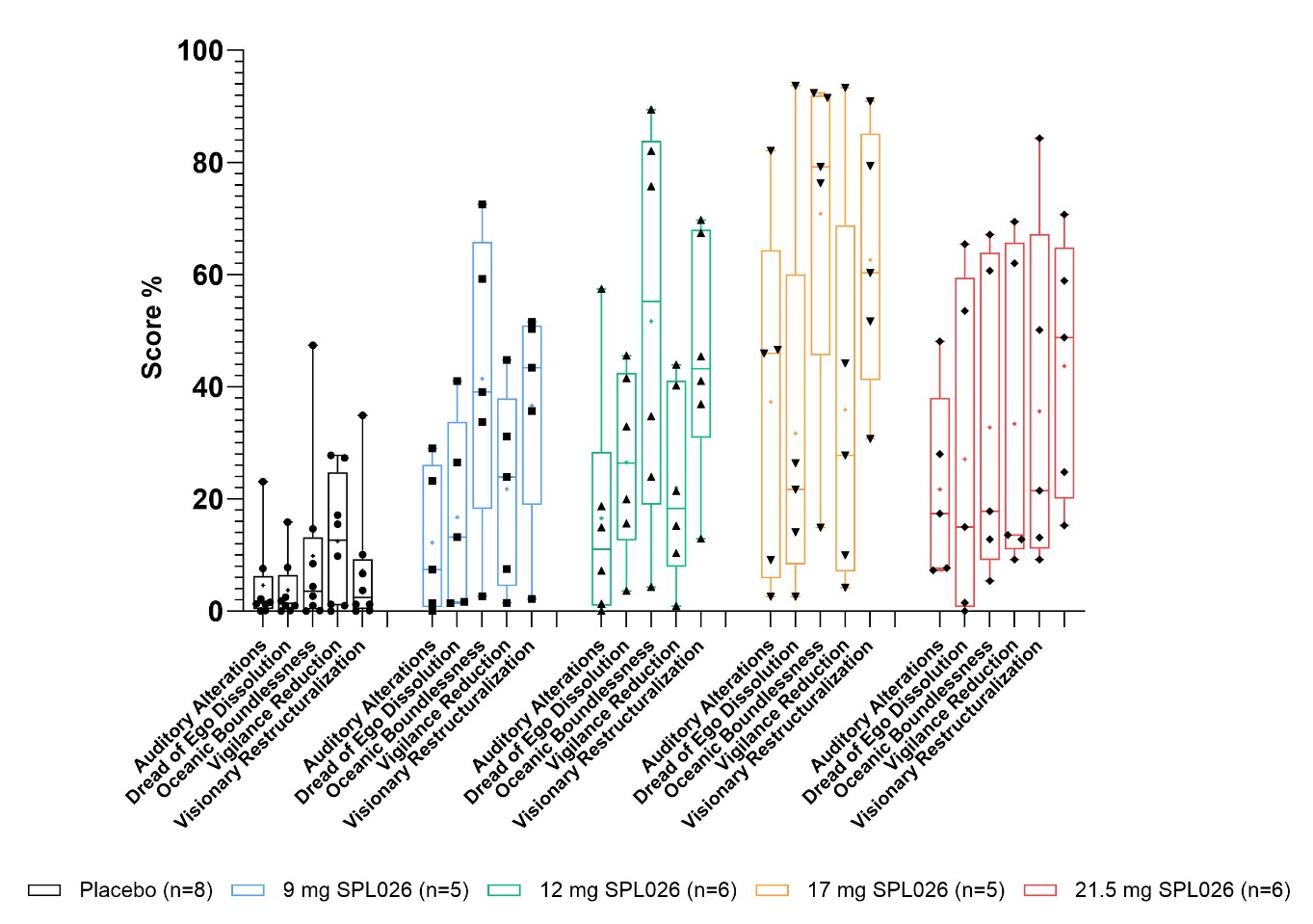
**

**A**

**
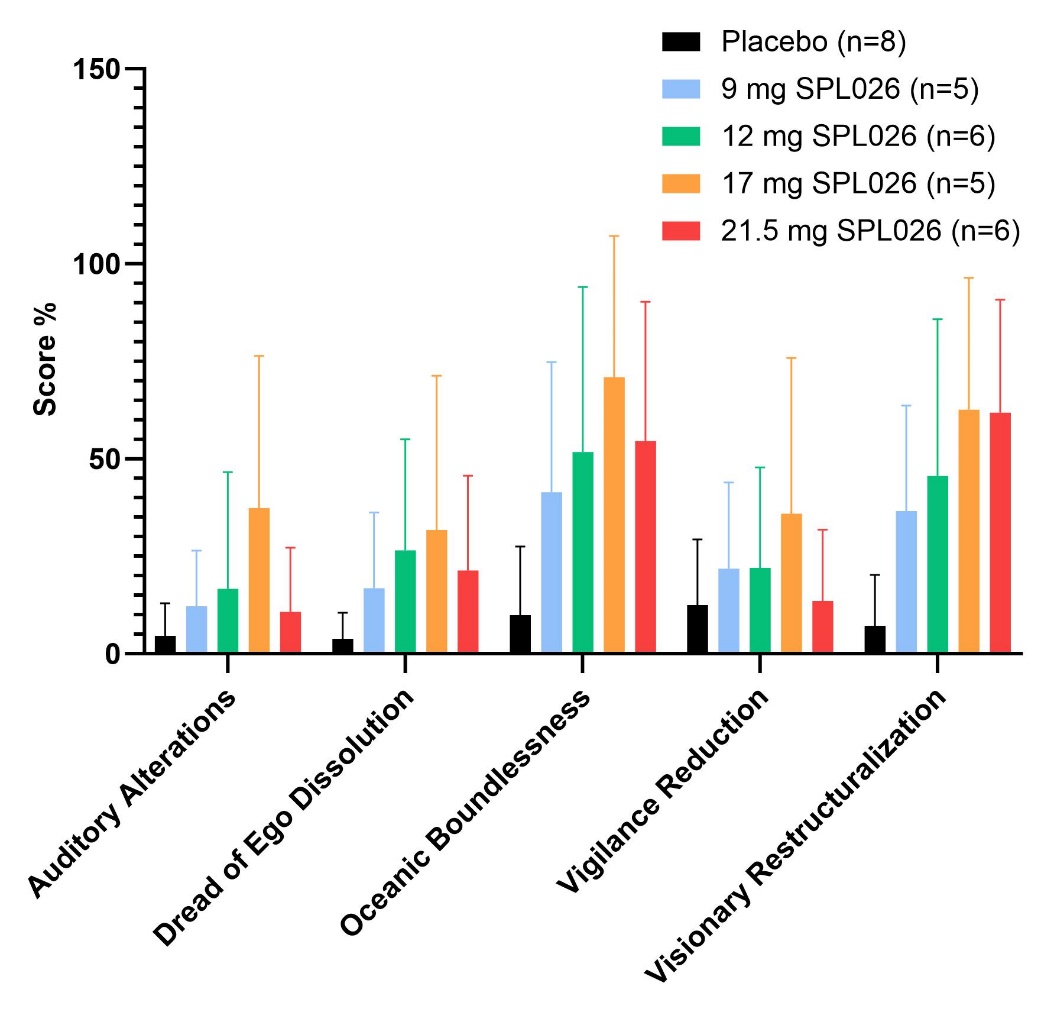
**

**B**

**
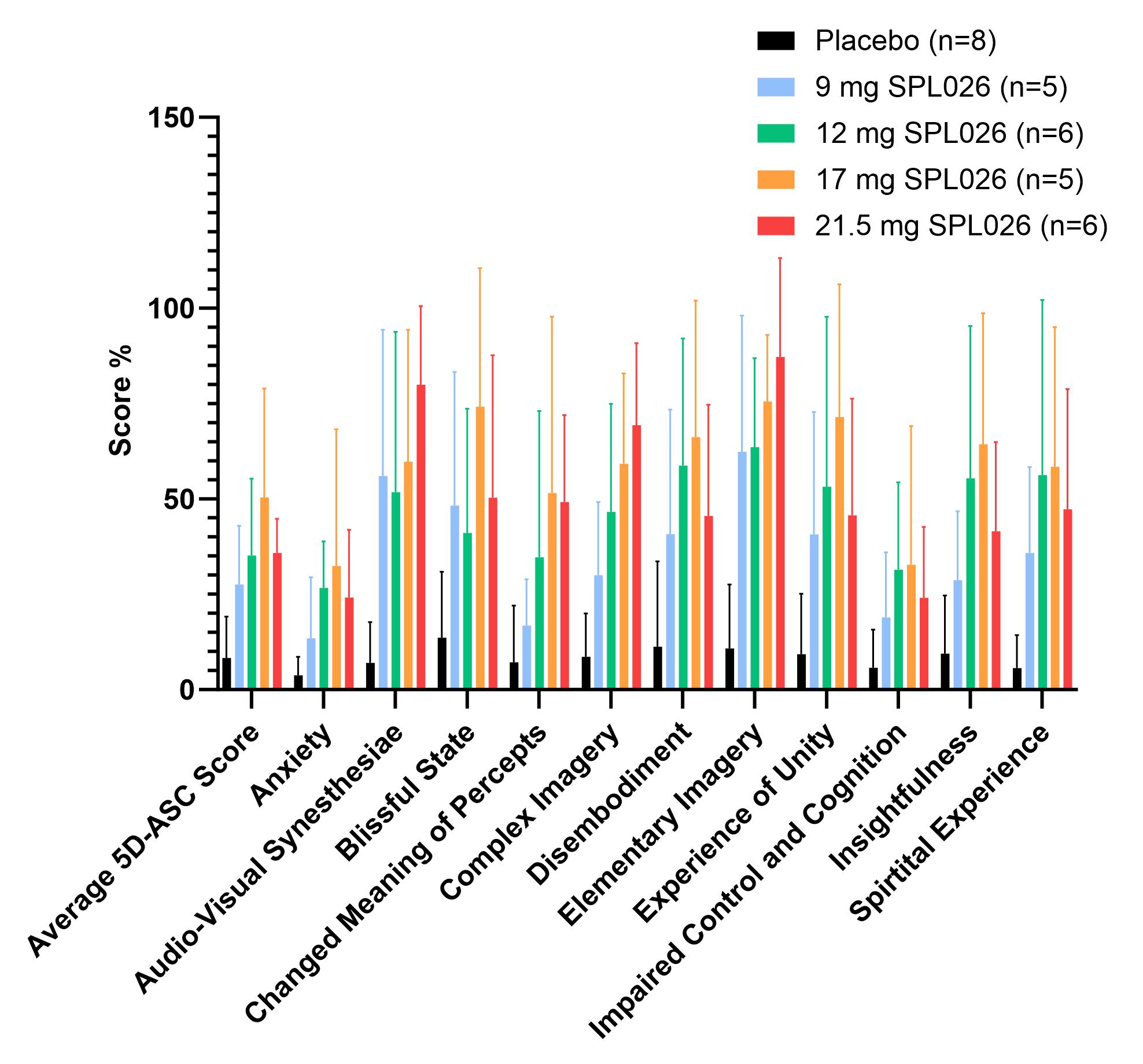
**

**C**

(A) Box and whisker plots of the mean score of each of the five dimensions of the 5D-ASC. Individual observations are shown as black shapes, the line through box is median and “+” is the mean. (B) Bar graph showing mean score of each of the five dimensions of the 5D-ASC. Error bars are standard deviation. (C) Bar graph showing mean score of each of the 11 subscales of the 5D-ASC. Error bars are standard deviation.

**Figure S2 Relationship between C_max_ and Acute outcome measures**


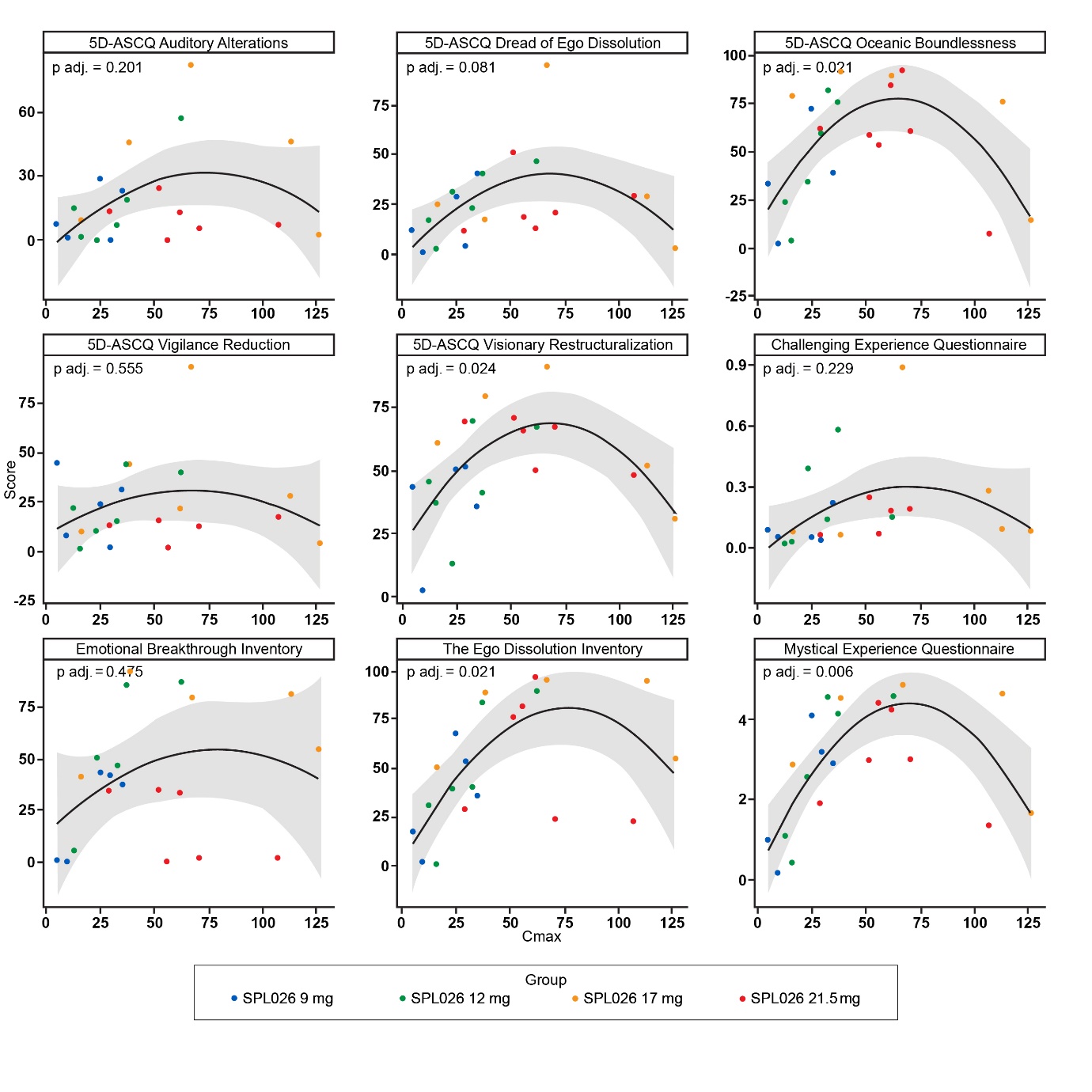


On the x-axis the C_max_ values are shown. On the y-axis the average scores on the different scales are shown. In the scatterplot, individual observations are shown as colored dots. The p-values corresponds to the quadratic regression analysis results after FDR correction, abbreviated as “p adj.”.

C_max_, maximum plasma concentration; 5D-ASCQ, 5-Dimension Altered States of Consciousness Questionnaire.

**Figure S3 Relationship between C_max_ and acute outcome measures, excluding 3 participants with C_max_ >80 ng/mL**

**
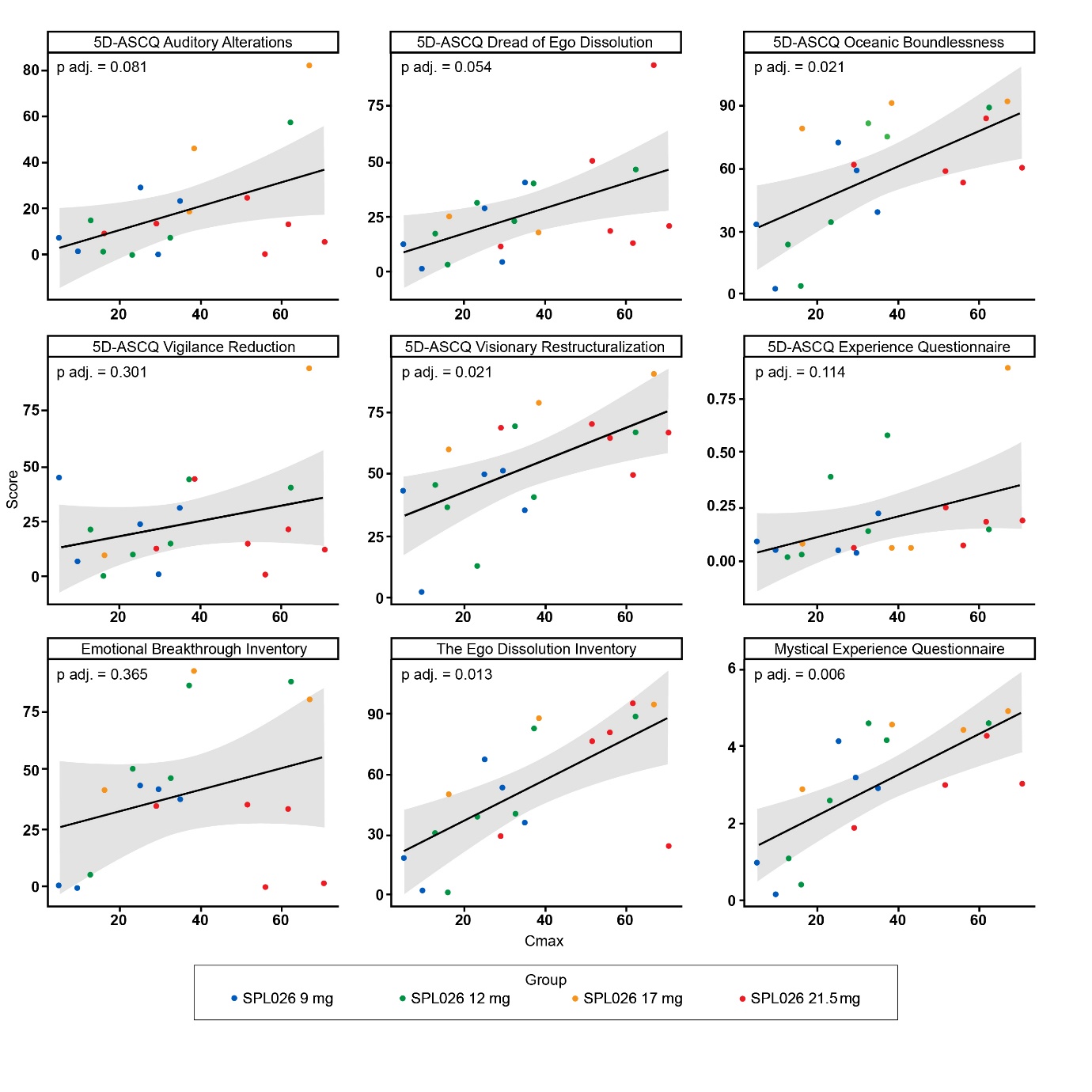
**

On the x-axis the C_max_ values are shown. On the y-axis the average scores on the different scales are shown. In the scatterplot, individual observations are shown as colored dots. The p-values corresponds to the Pearson’s correlation analysis results after FDR correction, abbreviated as “p adj.”.
